# Driver Adaptation to Partially Automated Driving in Urban Environments: Effects of Repeated Exposure and System Capabilities on Drivers’ Trust, Monitoring, and Response

**DOI:** 10.1177/00187208261460544

**Published:** 2026-06-18

**Authors:** Elena Malaika Nkusi, Jasmin Verena Schneider, Maximilian Wiegand, Klaus Bengler

**Affiliations:** 1 Chair of Ergonomics, TUM School of Engineering and Design, 9184 Technical University of Munich, Garching, Germany

**Keywords:** human-automation interaction, driver behavior, driver adaptation, accident analysis, attentional processes, reaction time

## Abstract

**Objective:**

This paper aims to quantify how the repeated use of partially automated driving (PAD) systems in urban environments influences drivers’ trust, monitoring, and intervention, and how exposure to explicit system limits affects emerging adaptations.

**Background:**

PAD is becoming increasingly available and expanding into urban environments. Meanwhile, safety depends on adequate driver behavior. Previous studies have reported decreases in vigilance and changes in trust with the repeated use of driver assistance systems. However, there is a general lack of studies examining driver adaptation to PAD systematically over time and in urban environments.

**Method:**

We conducted two driving simulator studies with a total of 45 participants driving repeatedly on an urban commuting route for five drives with intermissions of one or two days. We investigated the influence of mere exposure and exposure to explicit system limits. Both studies employed a multimodal measurement approach, combining self-reported and observational data.

**Results:**

Trust and single-glance aversions are moderated by system limits, alongside mere exposure. The number of critical responses to system failures reveals alarming driver behavior despite the use of a state-of-the-art driver monitoring system.

**Conclusion:**

Driver adaptation is highly event-driven as system limits calibrate trust, sustain monitoring, and improve intervention behavior. Moreover, state-of-the-art driver monitoring systems may not be sufficient to ensure the long-term safety of PADs in urban environments.

**Application:**

Urban PAD should adopt system limit warnings paired with gaze history-dependent prompts. Future studies and evaluations should extrapolate the revealed adaptation effects.

## Introduction

As of 2024, around 28% of newly registered vehicles worldwide were equipped with a partially automated driving (PAD) system, and adoption is rising (Berg Insight, 2025). In PAD, defined as Level 2 (L2) by the Society of Automotive Engineers (SAE), the system takes over both lateral and longitudinal control of the vehicle (SAE International, 2021). However, the monitoring task remains with the driver, who retains responsibility for driving. Accordingly, any potential safety benefit is contingent upon sustained driver monitoring. This is particularly the case in highly dynamic and complex scenarios, as occurs in urban environments, where anticipating critical situations and responding appropriately to takeover demands is crucial (Lehsing et al., 2016). Classic human factors work already cautioned that prolonged passive monitoring degrades vigilance and leads to out-of-the-loop performance problems (Bainbridge, 1983). Applied to PAD, such effects may impair drivers’ ability to resume control when a takeover is required. Additionally, a literature review by Frison et al. (2020) suggests that increasing experience with automated driving systems (ADS) can be expected to influence driver attitudes and lead to behavioral adaptations. Prior work suggests that system reliability is a key determinant of trust development (Lee & Moray, 1992). Applied to PAD, this implies that systems requiring only occasional intervention may foster increasing trust. With repeated use, this trust might grow to a level where drivers become overreliant on the ADS. Overtrust occurs when the expected system capabilities exceed the actual capabilities (Lee & See, 2004). These capabilities include aspects such as system reliability and functional limits within the operational design domain. In such cases, drivers may inadequately monitor both the driving environment and the system status, which can lead to safety-critical situations (Parasuraman et al., 1993). Recent evidence further synthesizes two issues in this respect: driver adaptation appears to be driven by the sequence and quality of key events rather than by the mere exposure duration (Nkusi et al., 2025), and longitudinal repeated-exposure studies involving multiple driving sessions per participant remain scarce (Frison et al., 2020), especially for PAD (Nkusi et al., 2025).

To address this gap and to explicitly investigate how system capabilities shape driver adaptation, we conducted a series of two driving-simulator studies. Manipulating system capabilities across studies enables them to be associated with observed adaptations. The studies investigate the development of drivers’ attitudes and monitoring behavior during the repeated use of PAD across a variety of urban scenarios. Our key contribution is to isolate the effects of system capability on learning-based adaptation in PAD across repeated drives in urban scenarios and to examine their safety-relevant consequences using a multimodal measurement approach.

### Related Work

A small number of studies have already investigated the repeated use of ADS and the diverse associated driver adaptation effects, for example, with SAE L3 (Dillmann et al., 2023; Kraus et al., 2020; Large et al., 2019; Metz et al., 2021) or with SAE L4 (Manchon et al., 2023). However, so far, only individual studies have been conducted on driver adaptation for PAD, as literature reviews by Frison et al. (2020) and Nkusi et al. (2025) highlight.

Gaspar and Carney (2019) provide initial insights into behavior during repeated use of PAD. This naturalistic driving study (NDS), conducted with 10 participants, examined differences in gaze behavior with and without an active PAD system. However, they did not explicitly observe or discuss behavioral changes over time, and no subjective measures were included. The authors emphasize that future studies should examine monitoring behavior during PAD in a more controlled manner and with larger sample sizes.

Complementing these observations, 4-week field trials on limited-access roads report that when PAD is engaged, the odds of distraction and hands-off episodes increase across weeks (Reagan et al., 2021, 2025). Notably, these were trials with PAD implementations from 2017, which differ from newer systems, for example, in terms of driver monitoring methods. Moreover, Fridman et al. (2019) collected large-scale field data of driving with driver assistance and PAD systems, the Advanced Vehicle Technology dataset (AVT-dataset). This dataset has the potential to enable the identification and quantification of driver adaptation effects. To our knowledge, so far, no published studies have investigated the AVT-dataset with a primary focus on driver adaptation across repeated PAD use.

Further studies investigated the driver adaptation at other automation levels. Thus, studies on lower automation levels, such as Beggiato et al. (2015), investigated attitudinal adaptation with an L1-system. The study was conducted as a field test with 15 drivers who completed 10 consecutive drives. The results reveal that the attitudes investigated, namely, trust and acceptance, towards the assisted driving system change with increasing experience with the system, with a point of stabilization occurring at the second (for acceptance) or fifth (for trust) drive. In particular, they determined that the course of these attitudes follows the power law of learning (Newell & Rosenbloom, 1981).

Other studies at higher automation levels (SAE L3/L4) were conducted, such as by Large et al. (2019). In the study, 49 participants completed drives across five consecutive days, with automation engaged during parts of the route. Their findings indicate that trust and acceptance towards the system increased with repeated use, even following an emergency handover.

It is worth noting that only a minority of studies in the context of human factors and ADS focus on urban environments (Frison et al., 2020). The urban environment can be described as more complex and is characterized by a high density of safety-relevant events. Specifically, this complexity arises from intense interactions with vulnerable road users, such as pedestrians or cyclists, and from occlusions in crossing situations, a high number of static objects, and complex, variable lane configurations and traffic rules (Götze et al., 2014; Krause, 2020; Lehsing et al., 2016; Twaddle et al., 2014). Such conditions compress temporal safety margins, for example, pedestrian conflicts, particularly with occlusions, shorten the time available to the first brake reaction (Lehsing et al., 2016; von Dewitz et al., 2024). PAD functions are increasingly available and are beginning to expand into urban environments. However, currently available PAD systems are not yet dedicated to cope with the diversity of urban environments and remain limited in scope, with requirements for reliable perception (e.g., detection of traffic lights or pedestrians) and stable behavior in dense urban scenarios identified (von Dewitz et al., 2024). Therefore, as systems mature, actual driver usage behavior and driver adaptation in urban environments become particularly relevant for safety and require specific investigations. Beyond empirical studies, international safety guidance by the International Organization for Standardization (ISO), such as ISO 21448 (ISO, 2022), emphasizes identifying and evaluating potentially unsafe scenarios. Thus, the principles are especially applicable for PAD in urban environments, where occlusions and complex traffic dynamics can create these scenarios.

Taken together, prior work that targets driver adaptation either investigates higher automation levels (Dillmann et al., 2023; Kraus et al., 2020; Large et al., 2019; Manchon et al., 2023; Metz et al., 2021), where monitoring demands differ fundamentally from PAD, or addresses solely attitudinal adaptations with L1 (Beggiato et al., 2015), leaving behavioral adaptation and safety implications untested. Others rely on naturalistic, observational designs in which exposure and intermission durations and the occurrence of key events were not experimentally controlled (Gaspar & Carney, 2019). Moreover, much of the PAD evidence comes from limited-access road contexts rather than urban environments (cf. Reagan et al., 2021; Reagan et al., 2025). This motivates a PAD, urban, repeated-exposure perspective that explicitly controls system capabilities using a multimodal measurement approach to investigate the co-evolvement of attitudes and behavior and analyzes intervention behavior in critical events.

### Driver Monitoring Systems

To ensure the drivers’ attention towards the driving task while using PAD systems, driver monitoring systems (DMS) are required for PAD under the UNECE type-approval framework for Driver Control Assistance Systems (DCAS). DMS play a crucial role in ensuring safety by detecting driver inattentiveness, issuing warnings, and attempting to restore attention. This is even more crucial against the background of potentially adverse driver adaptations due to repeated use of ADS. Earlier PAD systems were approved under UN Regulation No. 79 (2023) and typically relied on DMS with manual engagement monitoring (hands-on). In contrast, for new type approvals, UN Regulation No. 171 (2024) requires a DMS that detects visual disengagement based on the driver’s eye gaze, with head posture as a fallback. Thus, it allows hands-off operation when visually engaged. As a result, many PAD systems already on the road are legacy hands-on implementations, while new type approvals enable hands-off deployments. In addition, according to the Euro NCAP Safe Driving-Driver Engagement protocol (2025) driver monitoring performance as a total is scored, and there is no separate scoring for hands-on detection. Further, research suggests that hands-free systems may be equally effective in maintaining safety when combined with gaze-based monitoring (Josten et al., 2023). Given these, it can be expected that systems approved and available in urban environments in the future will increasingly support hands-off operations with gaze-based DMS, as applied in the studies presented here.

### Research Questions and Hypotheses

While existing research has explored driver adaptation effects during PAD mostly in single-session studies, little is known about how these effects develop across repeated exposures and intermissions, particularly in urban environments where a high density of key events compresses temporal safety margins. Notably, the role of system capabilities remains underexplored. To further understand the consequences of driver adaptation, it is essential to examine how these adaptations influence driver responses to critical events, such as system failures. To address these gaps, the present studies formulate the following research questions and hypotheses.

*Q1: What driver adaptation effects emerge during repeated use of a reliable PAD system in urban environments, and how do these effects evolve?* (**Study 1**)
  *H1.1: Trust in the PAD increases with exposure and stabilizes over time*.
  *H1.2: With increasing experience, the drivers’ monitoring behavior becomes more negligent*.

These hypotheses are based on the findings of Beggiato et al. (2015) and Large et al. (2019), presuming that the phenomena observed at other automation levels behave analogously to PAD.

*Q2: How do occurring system limits influence driver adaptation?* (**Study 2**)
  *H2: The development of trust and the drivers’ monitoring behavior differ depending on the occurrence of system limits*.

This hypothesis is formulated in an undirected manner, as two opposing arguments are considered. On the one hand, system limits may reduce trust and lead to improved monitoring behavior, as users acknowledge that the system cannot handle all situations. On the other hand, system limits could also lead to deteriorated monitoring behavior, as users might expect that the system is aware of its own constraints and will communicate any inability to handle specific situations, leading them to a higher trust and thus reliance on warnings instead of active monitoring.

*Q3: How do driver adaptations manifest in response to a system failure?* (**Both studies**)

## General Method

The two driving simulator studies share a similar setup in terms of the experimental environment, PAD system, and experimental track. The studies differ only with respect to one cross-study independent variable, namely, the presence of system limits (Study 2). The basic setup is described in this section, while the system limits are further detailed in Section Study 2. Both studies complied with the tenets of the Declaration of Helsinki and were approved by the Institutional Review Board of the Ethics Committee of the Technical University of Munich. Informed consent was obtained from each participant.

In both studies, participants were recruited via flyers and posters, social media channels (e.g., LinkedIn), and participant distribution lists for a two-step process. In the first step, participants completed a preliminary survey on demographic data. The eligibility criteria for the application include a driving license and no previous experience with PAD systems in urban environments. In addition, participants were exclusively permitted to participate in one of the studies. As males of student age predominantly completed the applications, in the subsequent second step, female and older applicants were preferably invited to the study in the interest of a balanced sample. The resulting samples are described in the respective studies’ section.

### Study Design and Procedure

Both studies applied a study design with repeated measures. Each participant drove in a driving simulator for 25 min in each of five (Studies 1 and 2) sessions and experienced a PAD system in an urban environment. Surveys, drives, and briefings resulted in a session duration of approximately 75 min in the first session and 45 min in all subsequent sessions. The intermission times between the drive repetitions of each participant were determined based on Ebbinghaus’s forgetting curve (Ebbinghaus, 1885), which suggests that the most significant memory decay occurs within approximately 30 hours. To ensure a balanced trade-off between feasibility and comparability, the intermission period was set as close as possible to this threshold. Thus, standardized intermissions of at least 1 day and a maximum of 2 days were implemented between the sessions. Consequently, if participants had their first session on a Monday, their second session was scheduled no earlier than Wednesday and no later than Thursday. This scheduling approach yielded participation durations of 9–13 calendar days. Figure 1 shows a schematic overview of the sequence of drive and intermission times and data collection intervals.

**Figure 1.**
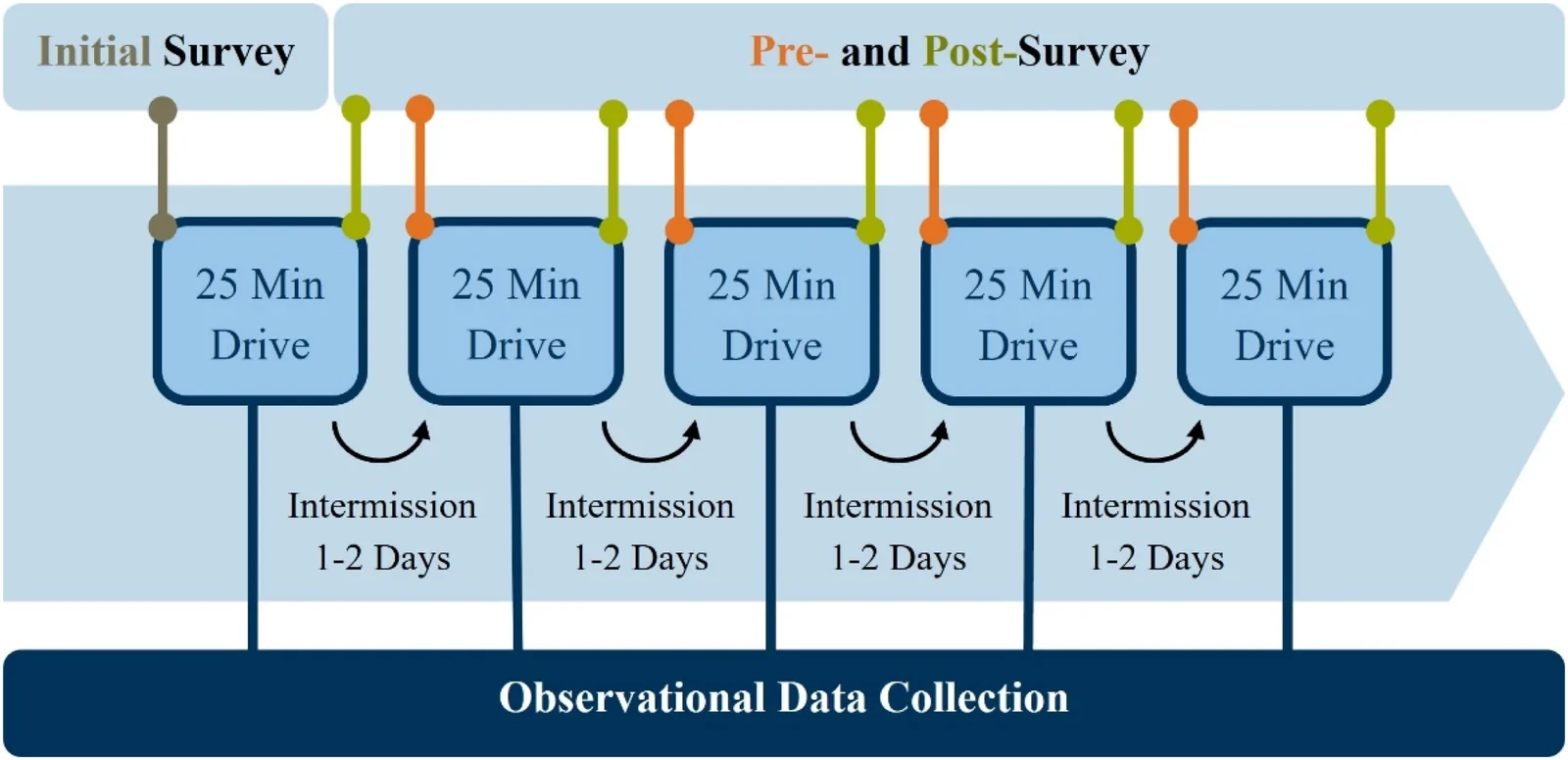
Schematic overview of the sequence of drive and intermission times and data collection intervals for the studies

The session procedure differed in the first session from the other sessions due to prior information and an initial pre-survey. Thus, before the first test drive, participants received training on ethics and safety procedures. Additionally, the option of withdrawing from the study without needing to cite reasons was outlined, and written consent was obtained. Following this, the participants completed the initial survey. There was a concise, written introduction to the system (see supplemental material). After the initial pre-survey, participants familiarized themselves with the simulator and the system in a 2-min drive. They were introduced to the system’s human-machine interface and followed the instructions of the experimenter to experience all options of driver-initiated system deactivation. The rest of the procedure was the same in all sessions. The participants were informed about the possibility of placing their cell phone in the cell phone holder, the tablet provided, and its applications. Subsequently, the experimenter calibrated the eye-tracking system. The calibration was verified according to the ISO 15007:2020 (ISO, 2020) before and after each test drive. Before the start of each test drive, the participants were again verbally informed about their responsibility to monitor the system and the environment. They were instructed to drive with the PAD system activated as far as possible and only to deactivate it if considered necessary due to discomfort with the driving situation or safety concerns. The test drive was introduced with a cover story being read aloud by the experimenter. This put the participants in the role of driving home after a busy day at work. They were asked to make their journey as comfortable as possible and to behave naturally. A follow-up survey was conducted after each drive. An additional debriefing was carried out in the last session when participants received an expense allowance of €75.

### Apparatus

Both studies were conducted in the static driving simulator of the Chair of Ergonomics at the Technical University of Munich (Figures 2 and 3). The simulator replicates a BMW E64 vehicle with an automatic transmission. SILAB 7.1 of the Würzburg Institute for Traffic Sciences GmbH (2019) serves as the driving simulation software. A high-quality, 6-channel projection system with a 60 Hz refresh rate provides an immersive environment. Three projectors are used for the front and back views. An additional sound system provides vehicle and environmental sounds. The simulator is equipped with an eye-tracking system with the Software SmartEye Pro 11.0 (Smart Eye, 2020). This monitors the driver’s gaze with three cameras in the interior and a fourth camera on the roof of the vehicle. A tablet with a quiz, radio, and video playback app, and a cell phone holder are within the reach of the driver.

**Figure 2.**
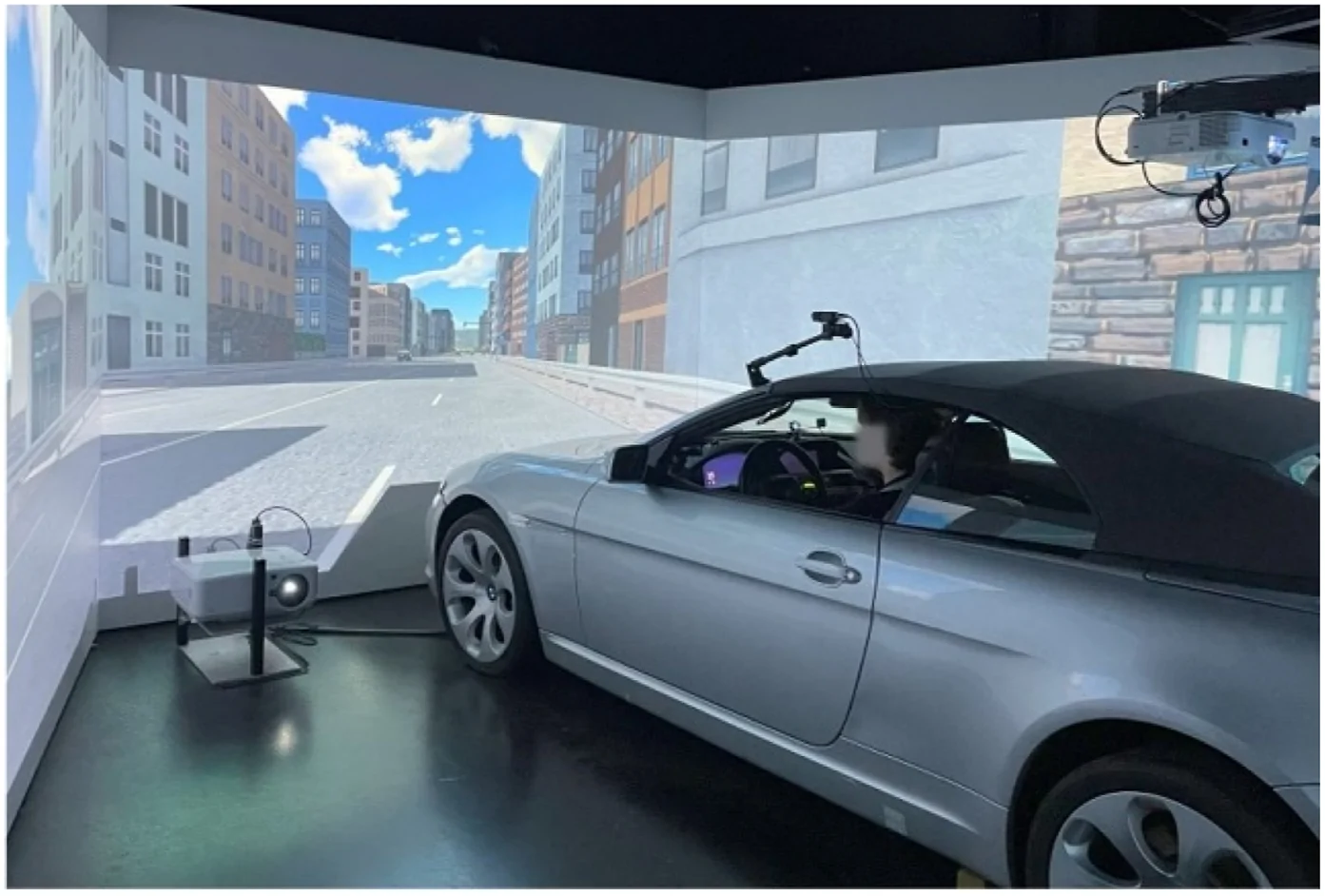
Exterior view of the driving simulator

**Figure 3.**
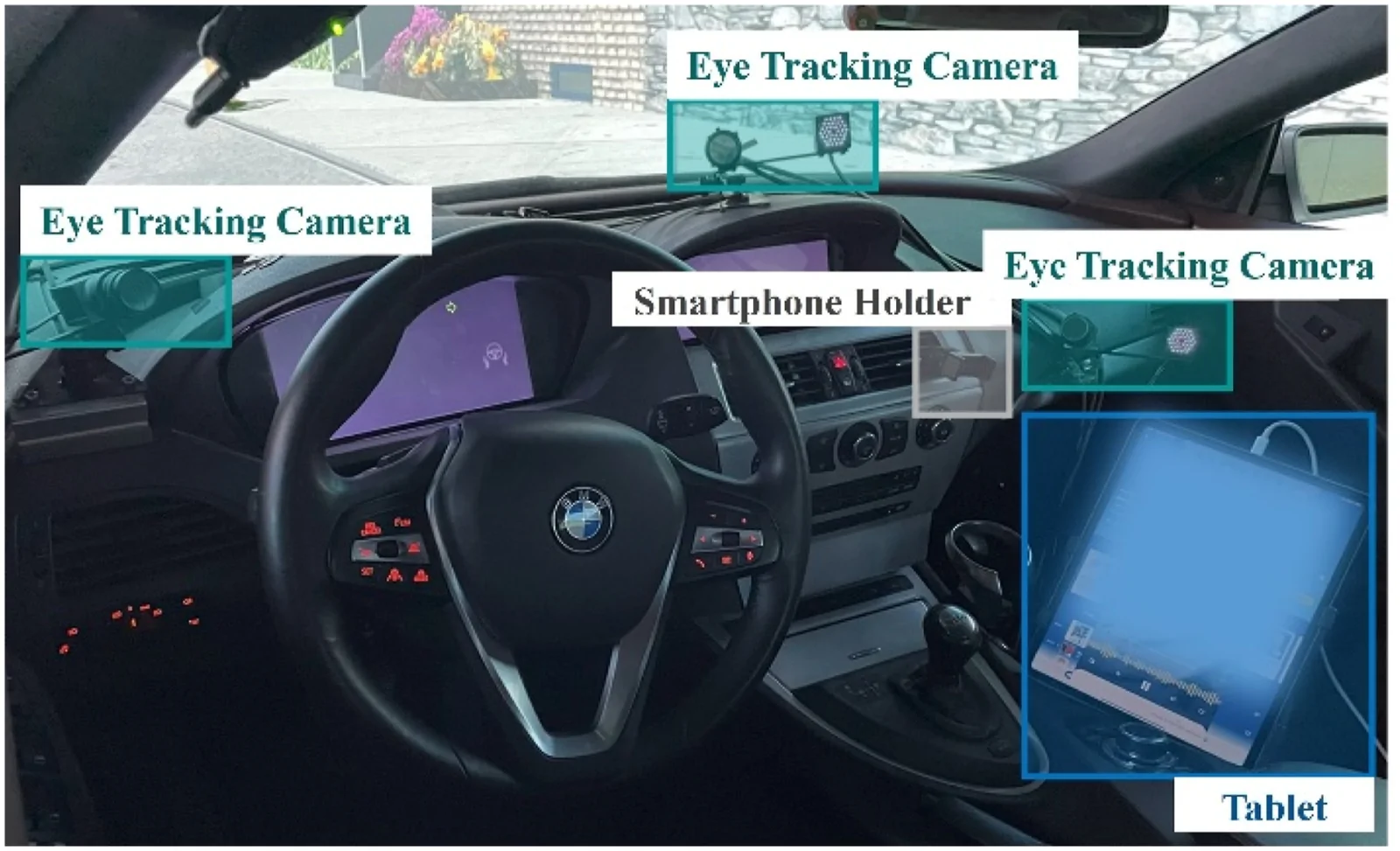
Interior view of the driving simulator

### Partially Automated Driving System

In both studies, the vehicle was equipped with a PAD system, which consequently provided both longitudinal and lateral control. The system could be activated and deactivated by pressing a button on the steering wheel. Additionally, deactivation was possible through steering interventions or braking. The system was highly reliable, functioning seamlessly across all scenarios while adhering to traffic regulations. It could respond appropriately to traffic lights, pedestrians, and road signs. However, in line with the experimental design, controlled system limits were implemented in Study 2, which are described in the respective study’s section. For clarity, in the present work, system limits refer to predefined operational boundaries that are explicitly communicated via a takeover request (TOR) and followed by a system disengagement as described below. In contrast, system failures refer to situations in which the system remains displayed as active and provides no warning, yet does not respond appropriately to the driving situation.

The instrument cluster was kept as basic as possible to avoid any confounding effect. Therefore, it contains only essential information about the current driving speed, the recognized speed limit, the specified target speed of the PAD system, and the indicator status. The system availability is shown by a steering wheel and a hands-free symbol, which are hidden when the system is not available, white when the system is available but inactive, and green when the system is active. The system was always available when driving at speeds greater than 5 km/h, except for the system limits. Each time the system status changes, a text box appears, which is displayed for 3 s to provide the information about the system status in text form, for example, “Automation activated.” In the event of system limits, a TOR in the form of a message appeared in the text box 4 s before system ejection with the inscription “System not available, please take over!” accompanied by an acoustic signal.

The DMS operated as a hands-free system, in accordance with current regulations and research results (see the subsection Driver Monitoring Systems). This design allows drivers to remove their hands from the steering wheel while the system is activated. However, the system monitors the driver’s gaze behavior and issues warnings if a continuous gaze diversion from the road center exceeds 5 s in accordance with UN Regulation No. 171 (2024) or if a cumulative gaze diversion of 10 s occurs within a 30-s time window in accordance with the Euro NCAP protocol (2025). Warnings are provided both visually via LED strips on the steering wheel and by changing the color of the instrument cluster and textbox displays, and acoustically, following a warning cascade with increasingly frequent alerts. The warning cascade can be interrupted and reset by turning the gaze to the road center for at least 200 ms if a single glance averted initially triggered the warning cascade, following UN Regulation No. 171 (2024) and 2000 ms if a cumulative gaze averted initially triggered the warning cascade, following the Euro NCAP Protocol (2025). The warning cascade is illustrated in Figure 4.

**Figure 4.**
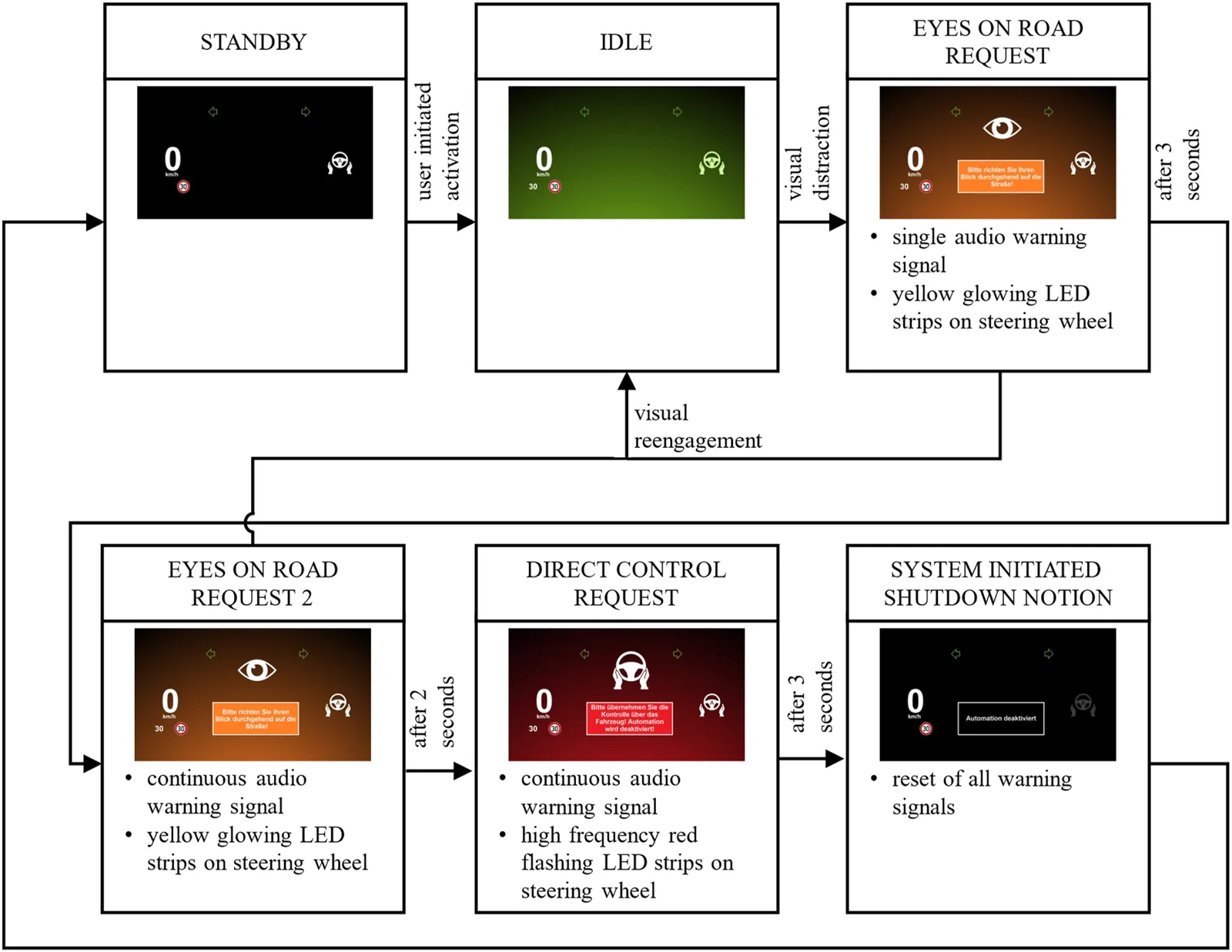
Transition diagram of the warning cascade. Arrows denote transitions governed by driver-monitoring and system logic. Under sustained visual distraction, states escalate from Idle to Eyes-on-Road Request, Eyes-on-Road Request 2, Direct Control Request, and finally System-Initiated Shutdown Notice. Visual re-engagement resets the sequence to Idle from either Eyes-on-Road Request state. Further system-modality changes for each state are listed beneath the corresponding display panels

### Experimental Track

The basic route described in this section applies to all studies and sessions. Differences between studies only concern the implementation of system limits. In addition, to simulate a daily urban commute realistically, there were slight differences in the occurrence of other road users between the sessions with comparable traffic densities.

The basic test track consisted of predefined scenarios that include sections of major and ring roads with speed limits of 50 or 60 km/h and residential areas with speed limits of 30 or 50 km/h, featuring parked cars, narrow lanes, pedestrian crossings, and cycling lanes. Furthermore, the route includes intersections with different right-of-way rules, signalized intersections, and different traffic densities. The supplemental material provides a list of all presented scenarios.

As the present work aims to investigate whether driver adaptations developed through repeated exposure lead to safety-critical intervention behavior in the event of a system failure (Q3), a system failure event was implemented. It occurred only in the final drive of each participant in both studies, after approximately 19 min of driving. In the event, the ego-vehicle approached a construction barricade in its ego-lane with a crane behind and a 10 km/h speed limit sign positioned 50 m ahead. The system did not respond to either the speed limit sign or the construction barricade by initiating braking or a lane change. A specific reaction reference line (RRL) represents the start of perceptibility of the system failure. Since the failure begins with the lack of the system’s response to the 10 km/h speed limit sign, the RRL was set at the longitudinal position of it. Thus, the time to collision (TTC) at the RRL when driving 30 km/h equals 5.79 s. Throughout the scenario, the system remained displayed as active until any driver intervention, and no warnings were issued. A schematic of the scenario is depicted in Figure 5.

**Figure 5.**
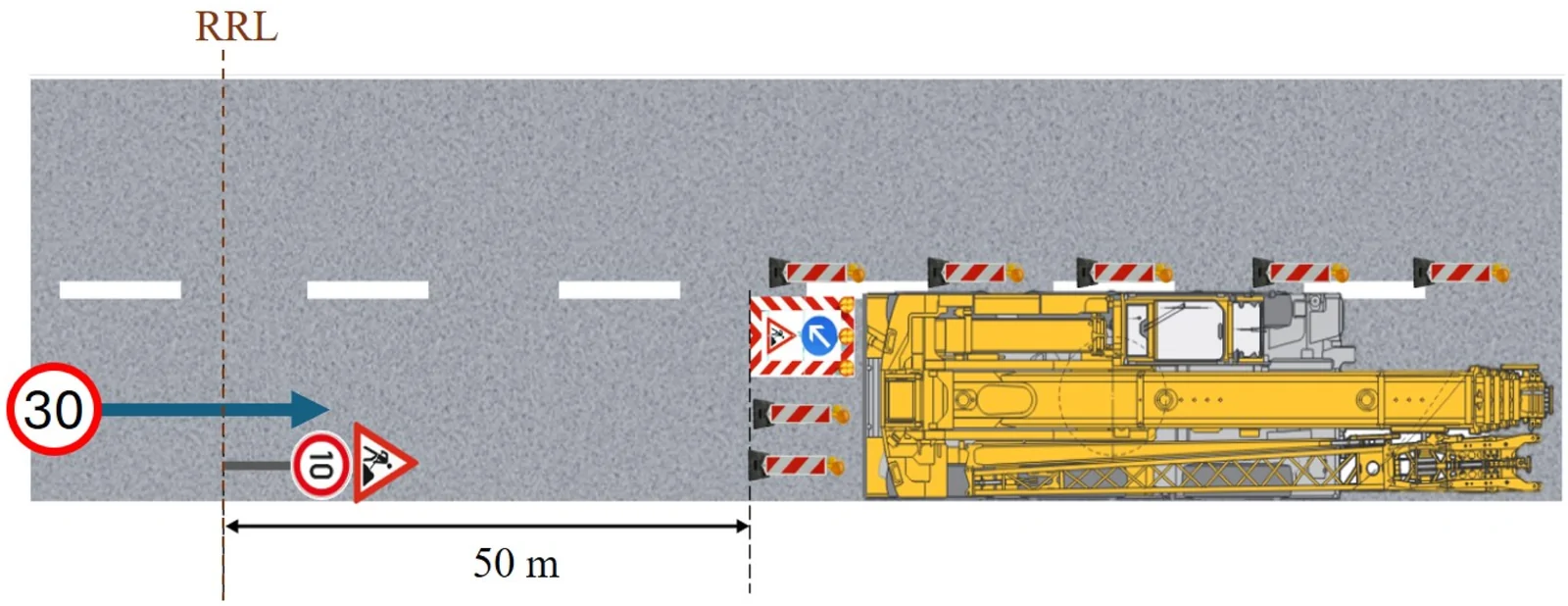
Top-view schematic of the system failure scenario, with a blue arrow indicating the ego vehicle’s driving direction and a brown dashed line depicting the reaction reference line

### Variables and Data Analysis

Given the general study design, the independent variables include drive repetition and, for self-reported data, the measurement time (pre- or postdrive) as within-subjects factors in all studies. Additionally, one between-subjects cross-study independent variable represents the occurrence of system limits (no limits vs limits). The dependent variables are both self-reported and observational.

Self-reported data were collected once before and once after each experimental drive using LimeSurvey (LimeSurvey GmbH, 2024). Self-reported data include trust in automation (TiA), measured with three subscales of the TiA questionnaire according to Körber (2019): Trust in Automation, Reliability/Competence, and Comprehensibility/Predictability. Given the limited number of items per subscale, analyses were conducted using an overall trust score, which was calculated as the mean of the subscales. The remaining subscales were excluded as they were not deemed applicable in this context. Additionally, measures of acceptance were surveyed, which Wiegand et al. (2025) described and evaluated. The full questionnaire is available in the supplemental material.

Observational data were recorded throughout the drive, including gaze, hand position, and driving data, which document system activation and deactivation, as well as the respective warning stage of the DMS. However, this paper focuses on gaze metrics and response to system failure events alongside trust data. Gaze data were converted into three metrics: Percentage Eyes off Road Time (PEORT) according to the ISO 15007:2020 (2020), Percentage Eyes on Instrument Cluster (PEC), and Single Glance off Road Duration (SGoRD), given that extended-duration glances have been linked to increased crash risk (Victor et al., 2015). The metrics were calculated based on pre-defined areas of interest (AOIs), in which the windshield was an AOI defined as the road center, and all other AOIs were considered averted gaze. A gaze turn to an AOI was only counted as such when a fixation (≥100 ms gaze duration (ISO, 2020)) occurred. The gaze metrics were only analyzed in the phases with activated automation. Additionally, to avoid distortions caused by the occurring system failure event, in the drive repetition with the controlled system failure event, only the gaze data up to the event occurrence was used for evaluation.

For intervention behavior in the event of a system failure, reaction times were calculated for each participant. The reference time for reaction time measurement was set for all participants to the time at which they crossed the RRL on the route. Accordingly, negative reaction times indicate anticipatory driver interventions occurring before crossing the RRL. In the case of non-intervention, reaction times were set to the maximum possible time until collision when driving according to the speed limit (TTC at the RRL). Additionally, the minimum TTC during the system failure event was calculated for each participant who intervened after crossing the RRL within an observation window extending from the RRL to either the first full stop or lane change. No TTC was calculated for participants who intervened before the RRL. For participants who collided with an obstacle, TTC was set to 0 s.

In addition to the independent and dependent variables, participant characteristics were assessed in the initial survey for sample description, which included age, gender, affinity for technology interaction (ATI) according to Franke et al. (2019), and propensity to trust according to Körber (2019). Further, previous experience with simulator studies (yes or no), previous experience with advanced driver assistance systems (ADAS) (self-developed items, scale from never heard of to use regularly), and driving regularity (from daily to less than monthly) were assessed. The supplemental material provides all questionnaires.

The data were processed and structured using MATLAB R2020b (2020) and statistically analyzed using R version 4.4.1 (2024). Please refer to the supplemental material for a complete list of R packages used in the data analysis. We analyzed the data with repeated-measures ANOVAs (rmANOVAs) and mixed ANOVAs to account for the repeated-measures (Study 1) and mixed (Study 2) structure of the observations (Field et al., 2012). Greenhouse-Geisser (GG) corrections were applied when Mauchly’s test indicated sphericity violations. All post-hoc tests were Holm-adjusted (Holm, 1979). Additionally, Mann-Whitney U was used instead of t-tests when normality was violated, but distributional shapes were comparable between groups, as assessed by Shapiro-Wilk tests and descriptives. When distributional shapes differed between groups, the Brunner-Munzel test was applied instead (Brunner & Munzel, 2000). A significance level of *α* = 0.05 accounted for all statistical tests.

## Study 1

The first study was conducted from March to May 2024. It examined repeated exposure to an urban PAD system without explicit system limits. This design primarily addressed *Q1* by investigating driver adaptation effects under a highly reliable system condition and provided a reference condition for analyzing the influence of system limits in Study 2 (*Q2*) and intervention behavior during the final system failure event (*Q3*).

### Sample

The recruitment procedure described in the General Method enabled a total of *n* = 30 participants to be invited to appointments. Due to non-attendance on at least one of the five appointments (*n* = 3) and technical problems (*n* = 5), the data sets of a total of *n* = 8 participants were excluded. This resulted in a final sample of *n* = 22 participants. The ages of the 4 females and 18 males ranged from 19 to 63 years, with a mean age of 25.95 years (*SD* = 9.29). For an overview of further sample characteristics, see Table 1.

**Table 1.** Overview of group distribution with data from both studies regarding sample size, age, gender, driving experience and regularity, simulator pre-experience, pre-experience with advanced driver assistance systems (ADAS), attitude for technology interaction (ATI), and propensity to trust

Measurement	Study 1 (Group 1, no limits)	Study 2 (group 2, limits)
*N*	22	23
Age	[19; 63] *M = 25.65* *(SD = 9.19)*	[18; 85] *M = 28.04* *(SD = 14.59)*
Gender	4 Female; 18 Male	3 Female; 20 Male
Driving license for >2 years	81.82%	86.96%
Driving regularity [daily, weekly, monthly, less]	13.64% Daily 36.36% Weekly 31.82% Monthly 18.18% Less	34.78% Daily 30.43% Weekly 21.74% Monthly 13.04% Less
Pre-experience with driving simulator studies	63.64%	34.78%
Pre-experience with ADAS (not urban)	50.00%	47.83%
ATI [(1) strongly disagree; (5) strongly agree]	*M * *= 3.88* *(SD * *= 0.60)*	*M * *= 3.68* *(SD * *= 0.62)*
Propensity to trust [(1) strongly disagree; (5) strongly agree]	*M * *= 3.02* *(SD * *= 0.61)*	*M * *= 2.81* *(SD * *= 0.57)*

### Results

#### Trust

Figure 6 illustrates mean trust scores and standard deviations across all five driving sessions, separated by measurement time (Pre- and Postdrive). The descriptives reveal slight increases in the mean values of trust during exposures and slight reductions during intermissions. Drive repetition five, in which the system failure occurred, is an exception, as exposure here leads to a reduction in the mean value of trust.

**Figure 6.**
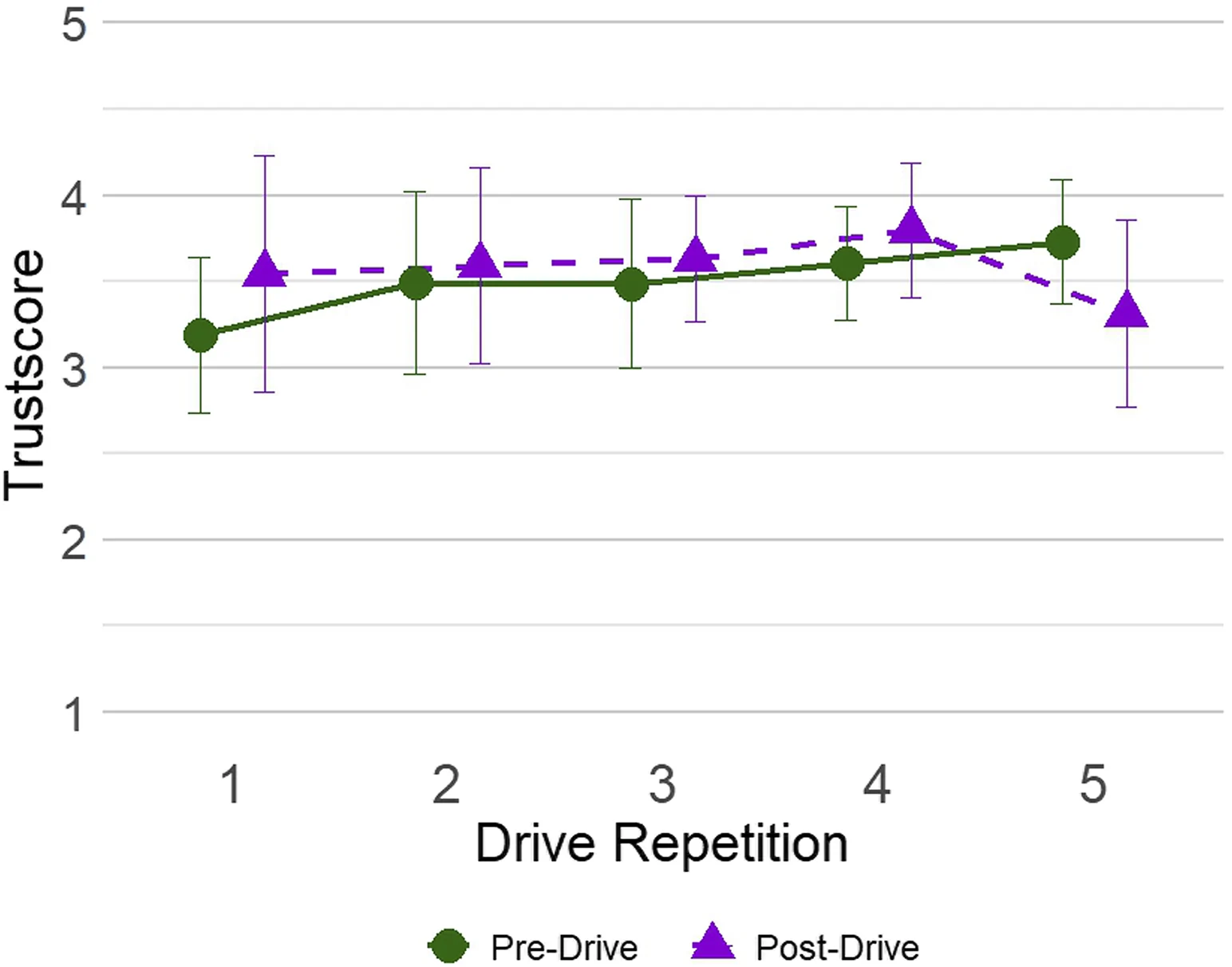
Mean Trustscores and standard deviations (error bars) before (green circles and solid lines) and after (violet triangles and dashed lines) each drive repetition (1–5). Data of Study 1.

A two-factorial rmANOVA (5 drive repetitions × 2 measurement times) revealed no significant main effect of drive repetition, (*F (2.52, 52.86) = 1.59, p = .21*) and no significant main effect of measurement time (*F (1, 21) = 2.49, p = .13*). However, the drive repetition × measurement time interaction was significant (*F (3.07, 64.46) = 9.05, p < .001,* GG-corrected*,* $\eta_{G}^{2}$ *= .07,* $\eta_{p}^{2}$ *= .30*). Thus, follow-up analyses of simple effects showed that for the pre-drive measurements, the effect of drive repetition is significant (*F (4, 21) = 6.30, p = .002*). Post-hoc tests with Holm corrections indicated that trust scores right before the first drive repetition were significantly lower than right before the fourth (*t (21) = −4.38*, *p = .002, d = −0.93*) and fifth drive repetition (*t (21) = −4.86*, *p < .001, d = −1.04)* with a large effect according to Cohen (1988). For the post-drive measurements, the main effect of drive repetition is not significant (*F (4, 21) = 1.64, p = .18*).

#### Gaze Behavior

For PEORT and PEC, Figure 7 presents means and standard deviations across drive repetitions. Consistent proportions of around 20% can be observed across drive repetitions, while a slight reduction in PEC can be observed.

**Figure 7.**
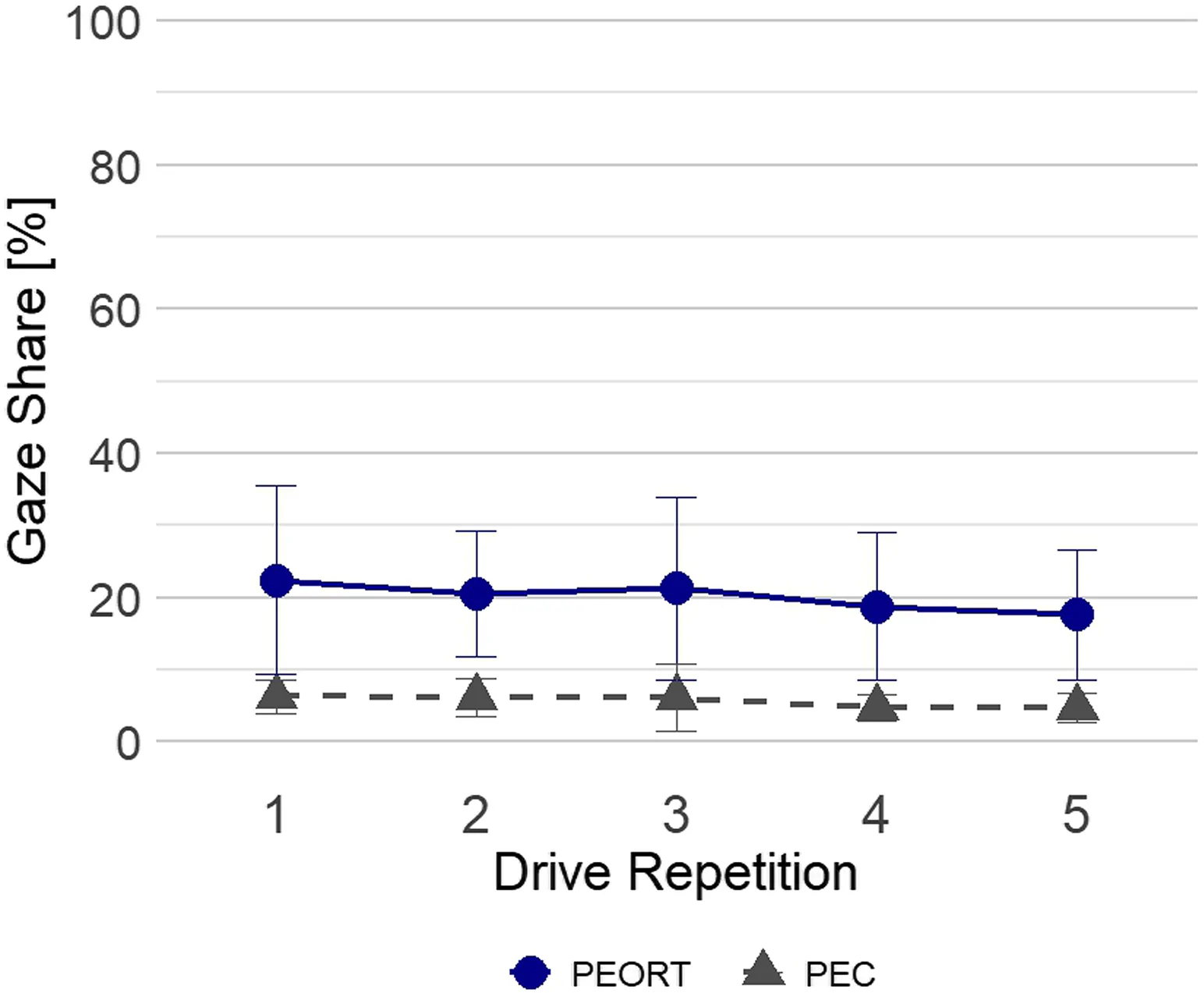
Mean Percentage Eyes off Road Time (PEORT, blue circles and solid lines) and Percentage Eyes on Instrument Cluster (PEC, grey triangles and dashed lines) and standard deviations (error bars) during activated automation times of each drive repetition (1–5), in drive repetition five up to the system failure event. Data of Study 1.

One-factorial rmANOVAs with the factor drive repetition revealed no significant effect on PEORT (*F (2.32, 48.68) = 1.35, p = .270,* GG-corrected), but a significant effect on PEC (*F (2.39, 50.19) = 3.21, p = .040,* $\eta_{G}^{2}$ *= .06,* $\eta_{p}^{2}$ *= .13,* GG-corrected). Holm-corrected post-hoc tests indicated a single significant difference between drive repetitions 1 and 4 (*t (21) = 3.36*, *p = .029, d = 0.717)*, reflecting a moderate effect.

Figure 8 illustrates SGoRDs as cumulative distribution functions (CDF). A general trend toward curve flattening with increasing repetition can be observed, indicating a shift toward longer gaze aversions over time. Notably, the curve for drive repetition five is steeper compared to drive four and strongly resembles drive repetition two, with drive repetition one (steeper) and repetitions three and four (flatter) contrasting more noticeably.

**Figure 8.**
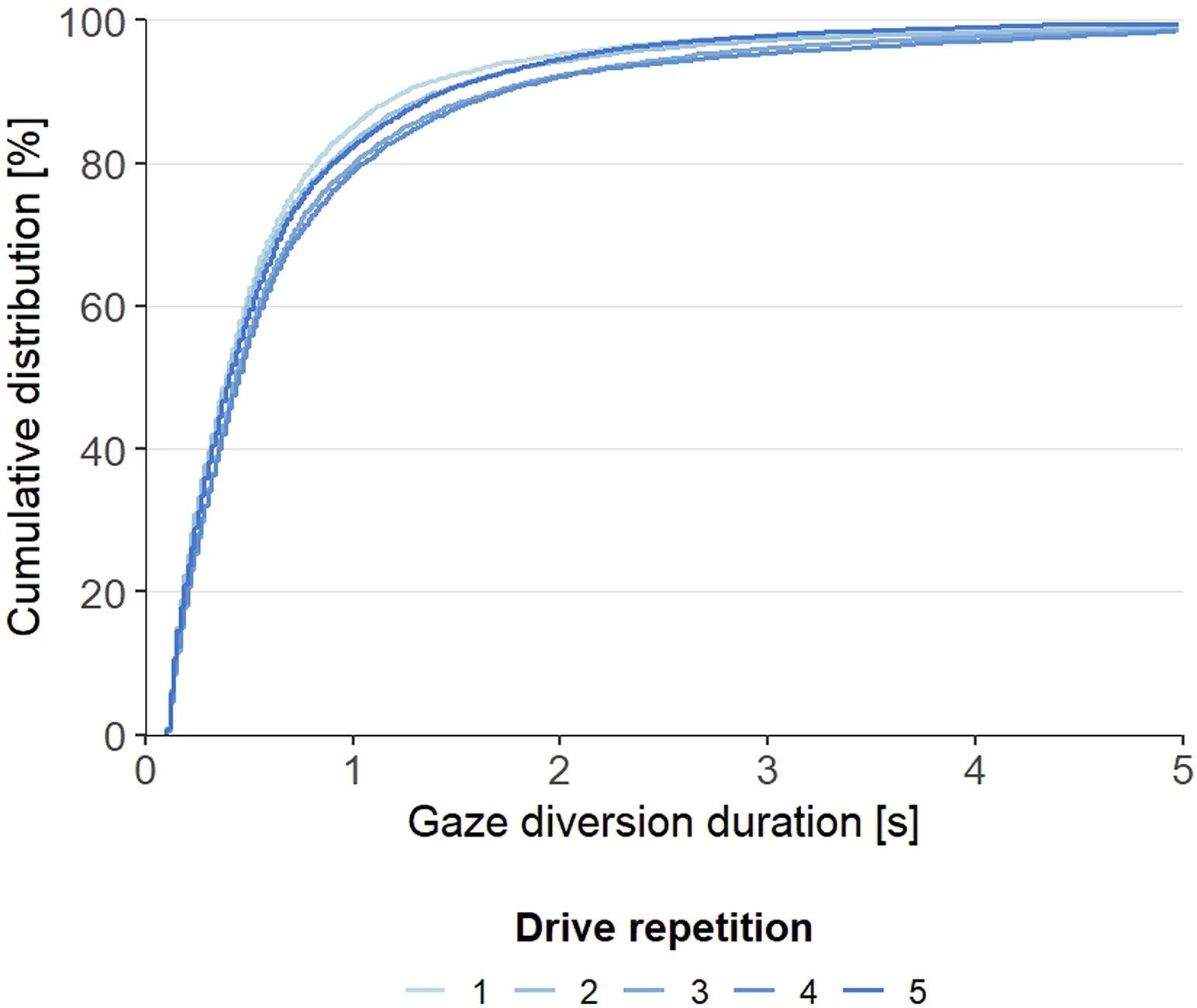
Cumulative distribution functions of single gaze off road durations across drive repetitions (1–5), in drive repetition five up to the system failure event. Curves show the proportion of diversions not exceeding duration t [s]. Data of Study 1

#### Response to System Failure

In the system failure event at the end of the final drive, notably, 86.36% of participants intervened only after passing the speed limit sign, thus exceeding the posted speed restriction. Minimum TTCs among those 86.36% were below the criticality threshold of 1.5 s (Grayson, 1984), for 42.11% (*n* = 8). Two participants (9.09%) collided with the obstacle in the system failure event. Descriptives are illustrated along with the subsequent study data (see Study 2).

### Discussion on Research Question 1

The results from Study 1 reveal that trust in a reliable PAD system showed a statistically significant interaction across repetitions, with small increases in early sessions and comparatively stable levels thereafter. Given the repeated-measures design, the significant simple effect of drive repetition on pre-drive trust should be interpreted cautiously, as post-hoc tests reveal that small session-to-session differences accumulated over time. Accordingly, the absolute changes between adjacent sessions were modest, although differences between early and later drives became more apparent. This is partly consistent with the findings from Beggiato et al. (2015), who similarly observed a rapid initial increase in trust towards an L1-system, reaching stability after five drives. In contrast to their study, which measured trust once per session, we captured trust before and after each drive. This allowed us to observe slight declines in trust during intermissions, particularly in early sessions. Notably, these intermission decreases became progressively smaller, while trust increases during exposure periods also became less pronounced over time, excluding the drive featuring the system failure. These findings do not contradict the results from Beggiato et al. (2015) but rather extend them, offering additional insights into the dynamics of trust formation and retention across intermissions of 1–2 days. Collectively, the results suggest that trust development in L1-systems and PAD systems likely follow similar patterns, with increasing stability across repeated exposures, thus providing partial support for *H1.1*.

Our findings regarding drivers’ monitoring behavior provided partial support for *H1.2*, which hypothesized increasingly negligent monitoring over time. While PEORT did not decrease significantly, more subtle indicators of adaptation were observable. Specifically, descriptive patterns suggest an increase in longer SGoRD proportions across drive repetitions. Although these changes were not statistically tested, they point toward a shift in gaze behavior that may reflect reduced supervisory monitoring. Notably, SGoRD curves in the fifth session returned to steeper patterns, resembling earlier drives. Here, a possible explanation could be participants’ heightened expectations or curiosity, knowing it is their final study session, anticipating something unusual occurring, given they had previously experienced nothing but a highly reliable system.

The nuanced findings align with results from Gaspar and Carney (2019) insofar as attentional changes were detectable through the SGoRD data rather than through the PEORT data and highlight the importance of examining detailed gaze metrics to detect subtle changes in monitoring behavior. However, our study additionally revealed apparent differences in gaze behavior across repeated sessions, which Gaspar and Carney (2019) did not observe, presumably due to the presence of system limits in their NDS.

In summary, addressing *Q1*, repeated exposure to a reliable urban PAD system leads to a pattern consistent with early increases followed by relative stabilization and subtle shifts in monitoring behavior, marked by longer SGoRD rather than in a PEORT increase, likely due to the forced gaze requirements of the DMS.

## Study 2

The second study took place from September to November 2024 and mainly addressed *Q2*. The study was implemented to examine the driver adaptations with the occurrence of system limits. Therefore, system limits were implemented, while the rest of the methodology remained identical to Study 1, including the system failure event in the final drive. A total of five system limits were presented in each of the five drive repetitions to ensure a comparable experience for participants across sessions. The limits were selected to maintain realistic consistency in system functionality. Thus, route-dependent limits appeared in every drive repetition, including two two-lane left-turn intersections and one give-way intersection. The route was not adjusted for these limits, but the system issued a TOR as described in the General Method. Other limits occurred only under specific conditions, for which the route was adjusted so that these conditions occur equally for each participant. These included starting from a complete stop at a red light, maneuvering around a postal vehicle within the lane, and, due to adverse weather conditions, such as heavy rain. Figure 9 provides an overview of the drive repetition-specific limits.

**Figure 9.**
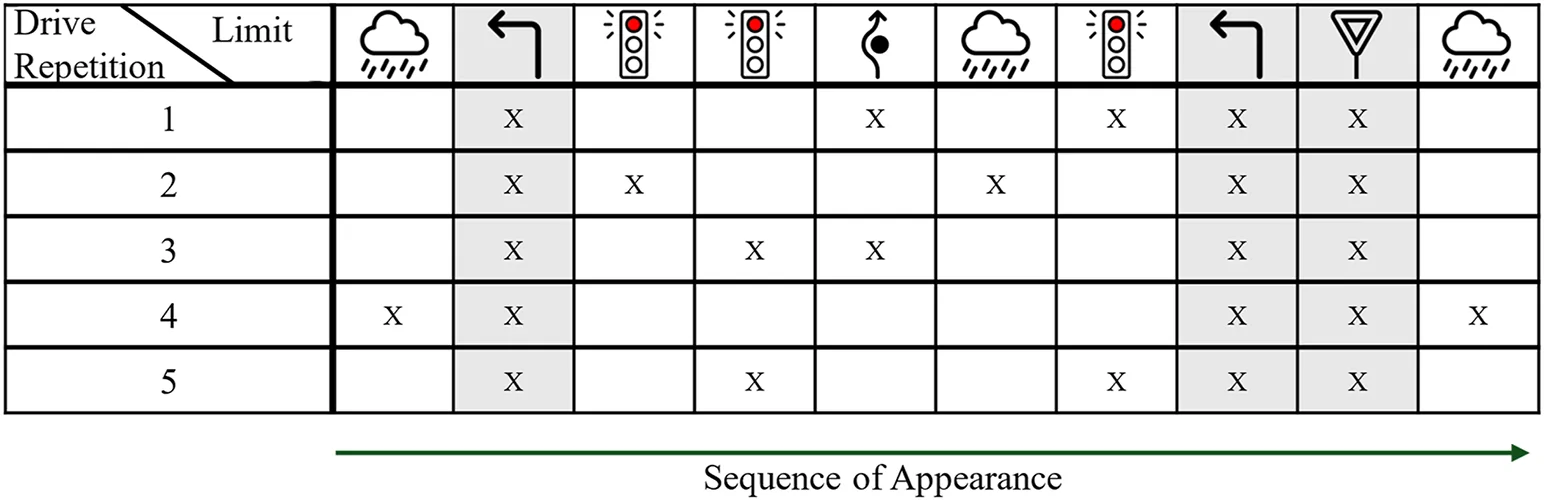
Overview of the occurrence of system limits in each drive repetition shown in order of appearance from left to right and with fixed system limits highlighted in gray. Occurring system limits are marked with X

### Sample

In Study 2, a total of *n* = 25 participants were invited. Due to technical problems (*n* = 1) and nausea (*n* = 1), the data sets of *n* = 2 participants were excluded. The final sample of *n* = 23 participants included 3 females and 20 males with ages from 18 to 85 years and a mean age of 28.04 years (*SD* = 14.59). For an overview of further sample characteristics, see Table 1.

### Results

#### Trust

Mean trust scores and standard deviations across all five driving sessions for participants in Study 2 are visible in Figure 10, separated by measurement time (Pre- and Postdrive).

**Figure 10.**
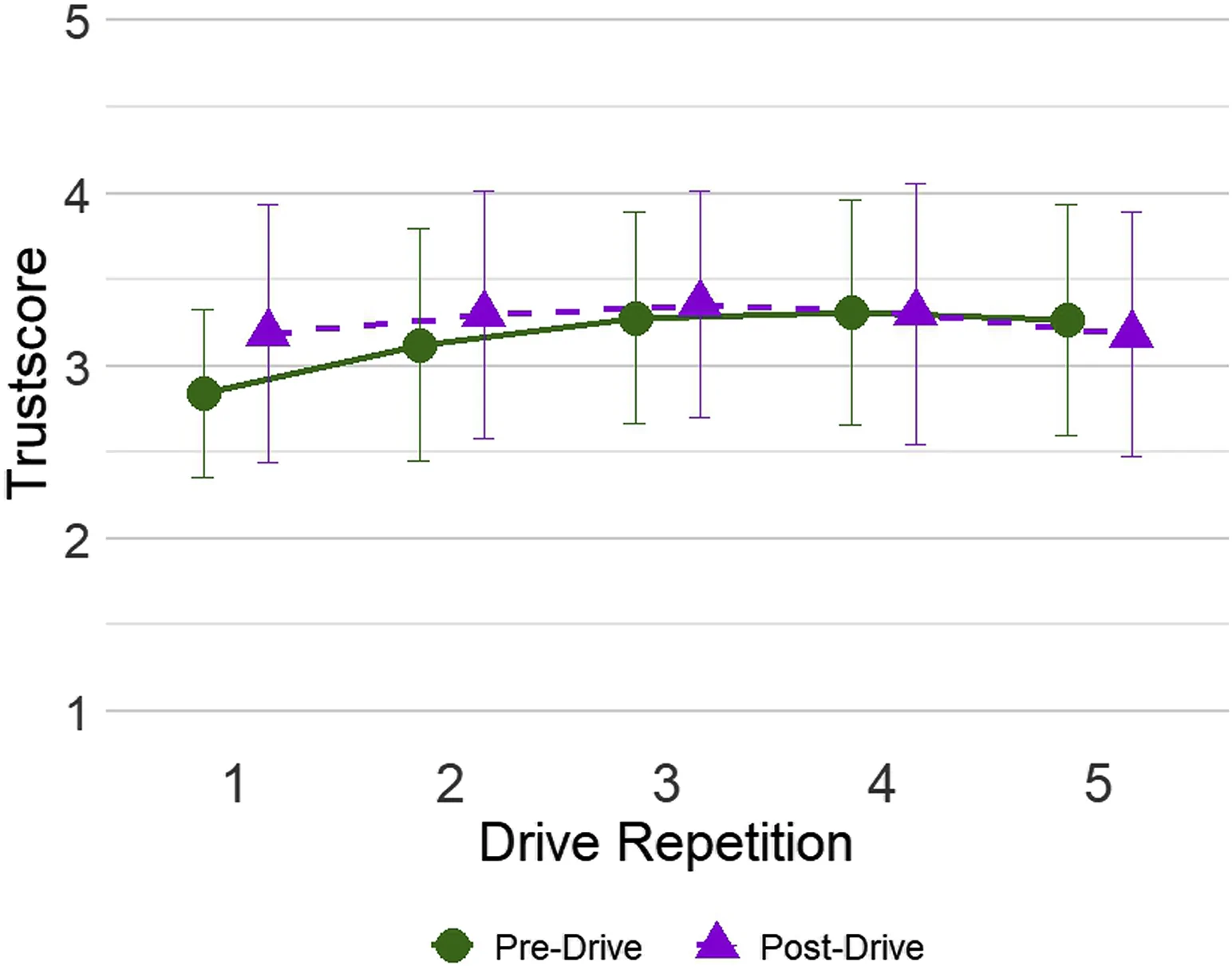
Mean Trustscores and standard deviations (error bars) before (green circles and solid lines) and after (violet triangles and dashed lines) each drive repetition (1–5). Data of Study 2.

The descriptives reveal comparable patterns to those observed in Study 1, showing slight increases in the mean values of trust during exposures and a slight reduction during intermissions, particularly in and after the first two drive repetitions. Drive repetition five, in which the system failure occurred, is again an exception, as exposure here leads to a reduction in the mean value of trust.

A three-factorial mixed ANOVA (5 drive repetitions, within × 2 measurement times, within × 2 Groups, between (no limits/limits)) showed a significant main effect of system limits (*F (1, 43) = 9.29, p = .004,* $\eta_{G}^{2}$ *= .073,* $\eta_{p}^{2}$ *= .178*) with higher trust in the group not experiencing system limits (*M = 3.54, SD = 0.50*) than in the group experiencing system limits (*M = 3.21, SD = 0.67*). Additionally, the main effect of drive repetition (*F (3.51, 150.8) = 3.09, p = .022,* $\eta_{G}^{2}$ *= .032,* $\eta_{p}^{2}$ *= .067*) and the main effect of measurement time (*F (1, 43) = 5.94, p = .019,* $\eta_{G}^{2}$ *= .006,* $\eta_{p}^{2}$ *= .121*) were significant, as was their interaction (*F (3.15, 135.37) = 9.19, p < .001,* $\eta_{G}^{2}$ *= .028,* $\eta_{p}^{2}$ *= .176*, GG-corrected). No further interactions were significant (all *p ≥ .094*). Consistent with the significant interaction, simple effects of drive repetition within the measurement times averaged over groups showed significant differences pre-drives (*F (3.53, 155.36) = 7.46, p < .001*) but not post-drives (*F (3.66, 160.83) = 2.16, p = .082*). The results of Holm-corrected post-hoc tests are depicted in Table 2. For reasons of conciseness, only significant results are included.

**Table 2.** Results of Holm-corrected post-hoc tests with *p*-values ≤.05. Signs follow the reported order (within: 1–4/1–5; between: no limits–limits). Effect sizes were calculated and interpreted according to Cohen (1988): small ≥0.20, medium ≥0.50, large ≥0.80

Measurement time	Contrast	*t (43)*	$p_{holm}$	*Cohen’s d (interpretation)*
Pre-drive	Drive repetition 1–3 (averaged over groups)	*−3.51*	*.008*	*−0.52 (medium)*
Pre-drive	Drive repetition 1–4 (averaged over groups)	*−4.49*	*<.001*	*−0.67 (medium)*
Pre-drive	Drive repetition 1–5 (averaged over groups)	*−4.86*	*<.001*	*−0.72 (medium)*
Pre-drive	No limits-limits (drive repetition 1)	*2.47*	*.017*	*0.74 (medium)*
Pre-drive	No limits-limits (drive repetition 2)	*2.06*	*.046*	*0.61 (medium)*
Pre-drive	No limits-limits (drive repetition 5)	*2.87*	*.006*	*0.86 (large)*
Post-drive	No limits-limits (drive repetition 4)	*2.72*	*.009*	*0.81 (large)*

#### Gaze Behavior

Figure 11 presents means and standard deviations of PEORT across drive repetitions for participants in Study 2. Descriptives of PEORT appear comparable to those in Study 1, as the data exhibit consistent proportions of around 20%. In contrast to Study 1, PEC values in Study 2 appear descriptively higher across drive repetitions.

**Figure 11.**
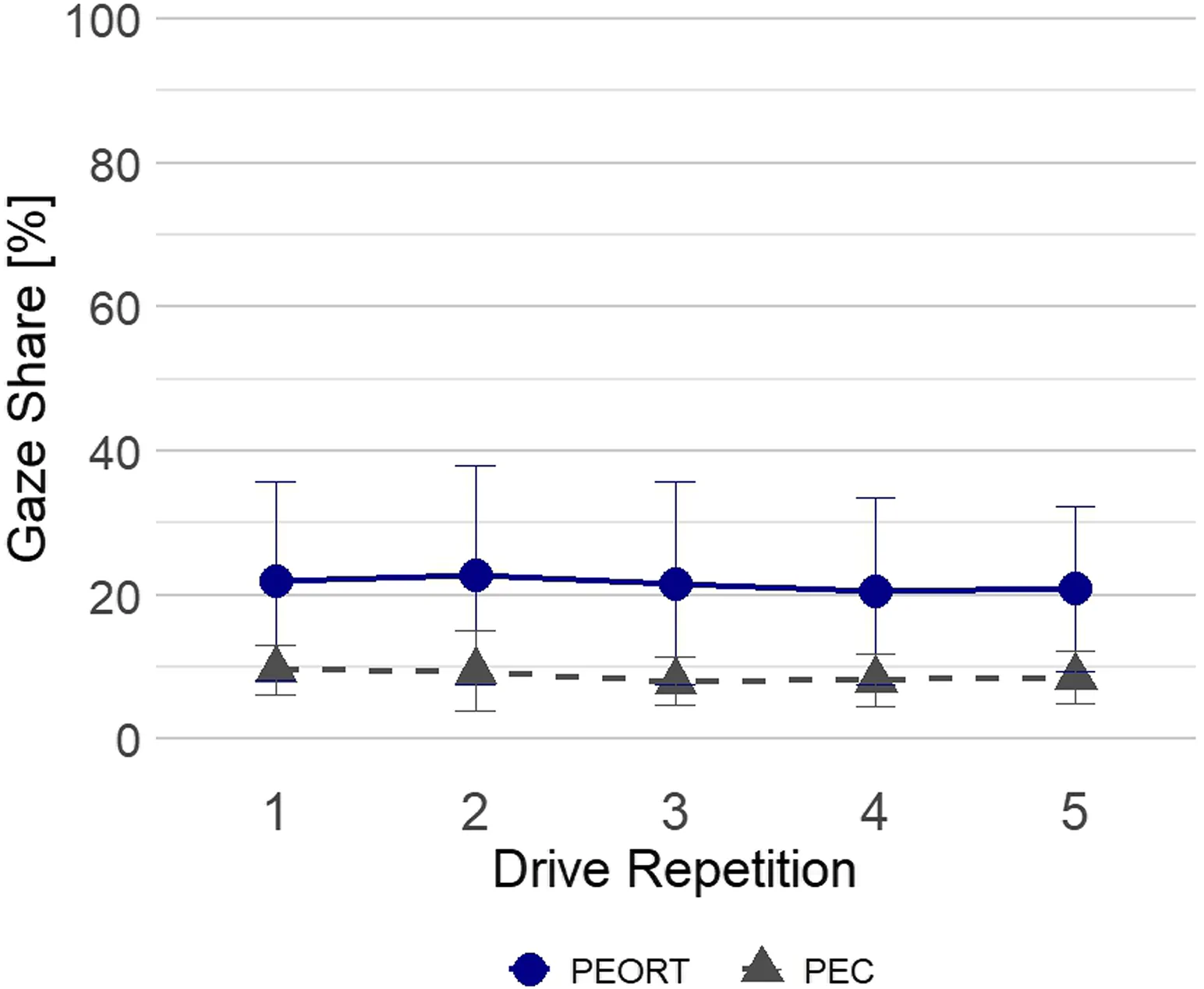
Mean Percentage Eyes off Road Time (PEORT, blue circles and solid lines) and Percentage Eyes on Instrument Cluster (PEC, grey triangles and dashed lines) and standard deviations (error bars) during activated automation times of each drive repetition (1–5), in drive repetition five up to the system failure event. Data of Study 2.

Two-factorial mixed ANOVAs (5 drive repetitions, within × 2 Groups, between (no limits/limits)) revealed no significant effect of drive repetition (*F (2.98, 127.96) = 1.86, p = .141,* GG-corrected) or occurring limits (*F (1, 43) = 0.19, p = .662*) on PEORT. In contrast, a significant effect of drive repetition (*F (3.01, 129.31) = 3.84, p = .011,* $\eta_{G}^{2}$ *= .028,* $\eta_{p}^{2}$ *= .082,* GG-corrected) and occurring limits (*F (1, 43) = 13.58, p < .001,* $\eta_{G}^{2}$ *= .176,* $\eta_{p}^{2}$ *= .240*) on PEC emerged with no significant interaction (*F (3.01, 129.31) = 1.37, p = .253,* GG-corrected), reflecting higher PEC values in the limits group. Holm-corrected post-hoc comparisons indicated significantly higher PEC values in the first drive repetition compared to the fourth (*t (43) = 4.05, p = .002, d = 0.60)* and fifth *(t (43) = 3.61, p = .007, d = 0.54),* corresponding to medium effect sizes (Cohen, 1988).

Figure 12 depicts SGoRD CDFs by drive repetition. The curves are tightly clustered and show only a modest rightward shift with increasing repetition, indicating a slight tendency toward longer gaze diversions. Pairwise separations are minor and most discernible for durations between approximately 1–1.5 s.

**Figure 12.**
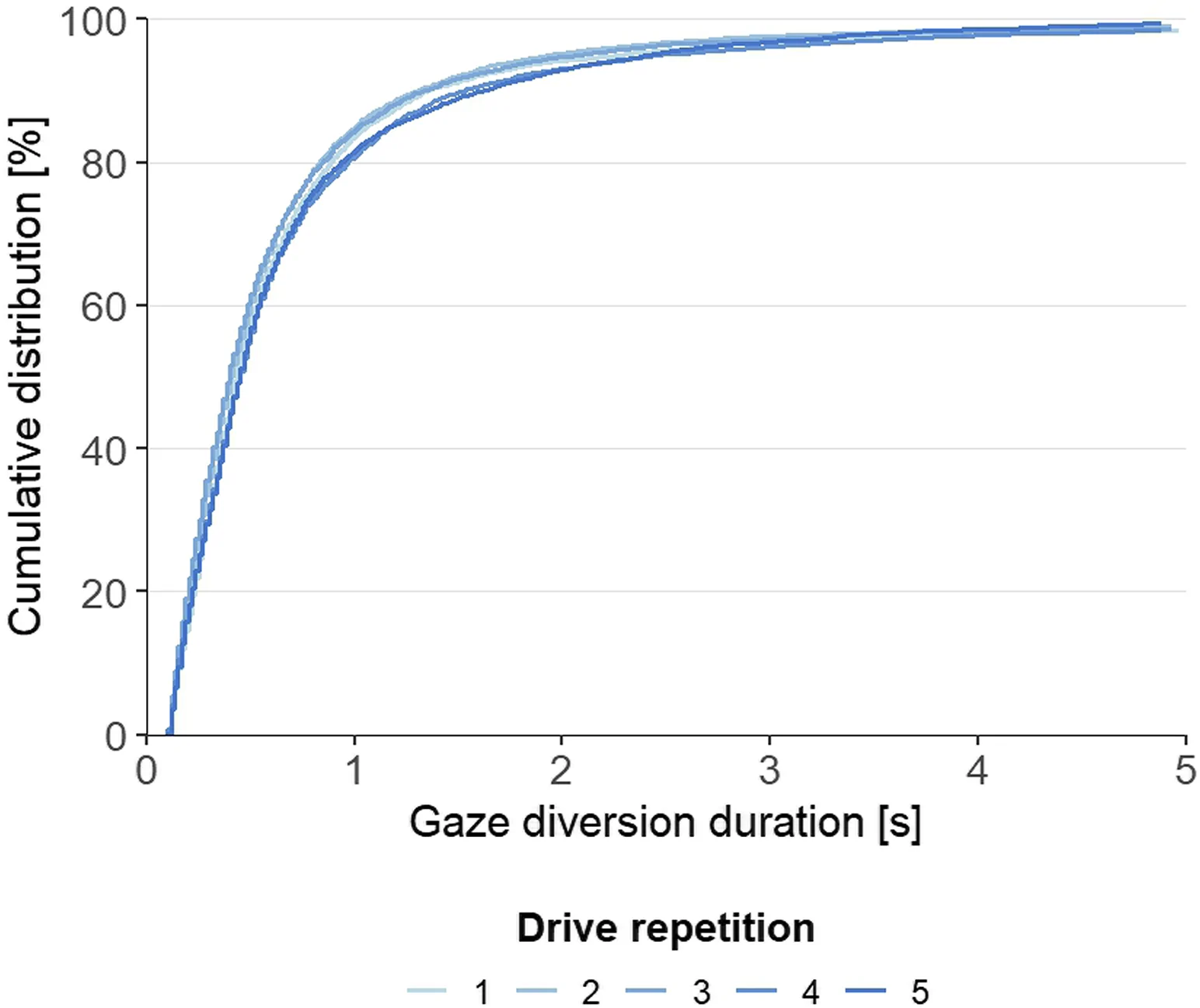
Cumulative distribution functions of single gaze off road durations across drive repetitions (1–5), in drive repetition five up to the system failure event. Curves show the proportion of diversions not exceeding duration t [s]. Data of Study 2

#### Response to System Failure

In Study 2, 65.22% (*n* = 15) of participants intervened only after passing the speed limit sign in the system failure event. Among these, *n* = 4 (26.67%) exhibited critical TTCs of <1.5 s (Grayson, 1984), which led to a collision for one participant. A Brunner-Munzel test revealed significantly lower reaction times in the group experiencing system limits (Study 2) than those in the group without system limits (Study 1) (*BM (42.67) = −2.13, p = .039*), with a medium effect size (*A*_*12*_ *= 0.67,* $CI_{95\%}$ *[0.51, 0.84]*) (Vargha & Delaney, 2000). Figure 13 shows reaction times and minimum TTCs for both groups with boxplots. The boxplots show that the reaction times for Group 1 (no limits) are noticeably longer, and a larger proportion of the participants who already violate the speed limit also have a minimum TTC below the criticality threshold.

**Figure 13.**
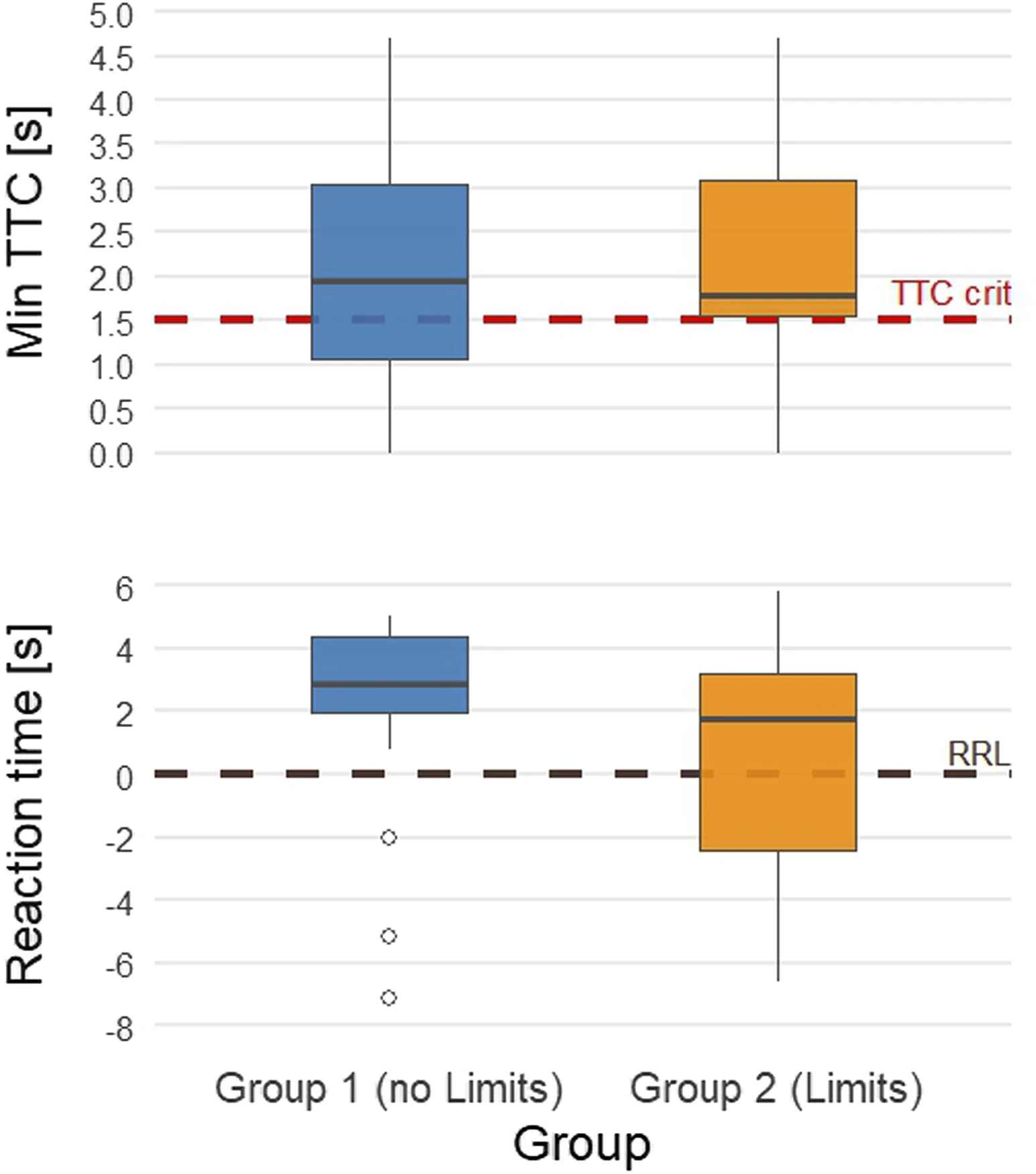
Reaction time and minimum time-to-collision (Min TTC) by group in the system failure event occurring in the fifth drive repetition for both groups. Min TTC [s] relative to the critical threshold of 1.5 s (TTC_crit, red dashed), including only participants who intervened after passing the reaction reference line (RRL). Reaction time [s] relative to the RRL (brown dashed). Boxes depict median and interquartile range (IQR); whiskers show 1.5×IQR; circles indicate outliers.

### Discussion on Research Questions 2 and 3

Regarding Q2, which asks about the influence of occurring system limits, the findings of Study 2 support H2 and indicate that system limits significantly influence driver adaptation. In particular, consistent with the significant main effect of system limits on trust, lower trust ratings were observed in Study 2 compared to Study 1, suggesting that drivers who encountered system limits remained more aware of the system’s constraints and, thus, their own responsibilities. At the same time, trust appeared less volatile, in particular, when encountering a system failure in the fifth drive repetition. Moreover, the occurrence of system limits was associated with better monitoring behavior, as indicated by increased PEC values. In addition, the cumulative SGoRD distributions suggest fewer long (e.g., >1.5 s) off-road glances in the limits condition, while PEORT remained comparable to Study 1. This might be caused by the drivers being more involved in the driving task due to the takeovers, which would be in line with the out-of-the-loop performance problem described by Bainbridge (1983). Thus, the presence of system limits appears beneficial in calibrating user expectations and preventing overtrust. This finding is consistent with prior work showing that users calibrate their trust based on their experience with system performance and reliability (Lee & Moray, 1992). A comparable pattern emerged here across repeated use. Notably, Large et al. (2019) investigated trust before and after a single TOR with a lead time of 10 s and found no significant effect on trust. In contrast, in our study, participants encountered multiple TORs per session with shorter takeover times (4 s). This higher frequency and urgency may explain the significantly lower trust ratings observed compared to the group without limits.

The data from both studies also allow for a differentiated answer to *Q3*, which asks how driver adaptations manifest in the event of a system failure. The results show that the influence of system limits on driver adaptations, previously reflected in the significant main effects on trust and PEC, is also reflected in the resulting intervention behavior. Participants show significantly shorter reaction times during a system failure event, with a medium effect size, if they have previously been confronted with system limits. In addition, fewer participants exhibited critical TTCs, and fewer collisions occurred in the limits condition. Nevertheless, it should be noted that the system failure presented was a highly salient and previously unencountered situation in which the system did not respond appropriately to a 10 km/h speed limit sign at an initial TTC of 5.79 s. Thus, the failure event can be considered controllable for attentive drivers. However, collisions still occurred in individual cases, and critical TTCs were observed for a considerable proportion of participants. This indicates that safety-critical manifestations of driver adaptation can emerge even after a small number of non-critical PAD experiences.

## Overall Discussion

In general, the results from both studies show driver adaptations in trust and monitoring during repeated urban PAD use. Interestingly, behavioral evidence goes beyond mere attitudinal adaptations. Across repeated drives, trust increased, and monitoring patterns slightly shifted over time. While PEORT remained stable, descriptive patterns in SGoRDs and the significant effect of drive repetition on PEC suggest subtle adaptations in supervisory behavior. In addition, participants exposed to explicit system limits maintained significantly lower trust levels and exhibited higher PEC values compared to the no limits group. In the system failure event, participants who had experienced explicit system limits across repeated drives showed shorter reaction times and fewer critical TTCs compared to those in the no limits condition. This indicates the observed driver adaptations manifest in safety-relevant behavior. At the same time, the presence of system limits did not eliminate critical cases entirely, suggesting that calibration effects improve but do not fully safeguard intervention performance in dense urban scenarios. Taken together, these findings align with Nkusi et al. (2025) in emphasizing that the perceived quality of usage factors, such as explicit system limits, emerge as key drivers of driver adaptation.

Notably, potential adaptations in PEORT may have been constrained by the gaze enforcement of the applied DMS. As a result, adaptations appear mainly in SGoRDs and PEC rather than in PEORT. Despite the supposed effectiveness of the DMS with regard to PEORT, the collision counts are concerning, once higher market penetration and broader availability of PAD in urban environments are considered. However, current DMS requirements are not explicitly tailored to dense urban settings, and the example implementation of a DMS applied here did not achieve the level of protection needed, which is noticeable in the critical TTC rates of up to 42%. As systems improve, fewer explicit system limits will likely occur, which impacts driver adaptations and, with repeated exposure, increases the chance that rare system failure events become safety-critical.

Overall, a coherent picture emerges. Repeated PAD use leads to adaptations in trust and monitoring, while explicit system limits recalibrate these adaptations and improve intervention behavior. Open issues and limitations are summarized in the following subsection. The conclusions for design and validation follow directly from this and are summarized in the subsection Implications and Future Work.

### Limitations

Despite the insights gained, our study has several limitations. First, the composition of participants may limit generalizability. We recruited a relatively homogeneous group of younger, predominantly male drivers who might not represent the broader driving population. Furthermore, the study was conducted in a controlled urban driving context using a driving simulator, which cannot fully capture the complexity and unpredictability of real-world urban driving. Moreover, regarding the DMS, the implementation used in our study was intentionally designed to reflect realistic future deployment in urban PAD systems based on current regulatory proposals. Unlike other studies that often employ no DMS or only manual engagement monitoring, our implementation included visual engagement monitoring aligned with current regulatory proposals (see the subsection Driver Monitoring Systems). While this supports the ecological validity of our findings, it may also have attenuated some adaptation effects, particularly in PEORT, which were constrained by the DMS’s enforcement of gaze compliance. These limitations suggest caution in over-generalizing the results. They also highlight opportunities for further research to verify how well our findings hold in more naturalistic, long-term settings.

### Implications and Future Work

#### Implications for Validation and Driver Modeling

Building on our results, repeated use rather than single sessions should guide study design and validation. Where the number of sessions is limited, potential adaptation trends should be extrapolated and reflected in the interpretation of outcomes. Furthermore, beyond exposure duration, event history needs explicit representation. The appearance and frequency of occurring system limit shape later intervention. Future cognitive driver models should therefore include the experience of certain events and exposure duration. This view is consistent with recent guidance that treats within-person usage over space and time as central and other influences as moderators (Nkusi et al., 2025). Driver modeling should carry these factors forward. Perceived quality of usage variables, such as experienced limits, update internal driver state across exposures and inform predictions about monitoring and response behavior. As highlighted by Nkusi et al. (2025), our results also point to the need to model these influences over time and to link attitudinal change with concrete behavioral outcomes to enable targeted countermeasures of safety-critical behavior. Accordingly, from a validation perspective, the results indicate that single-session evaluations of PAD systems are insufficient to capture adaptation effects. Safety assessments should therefore incorporate repeated-use scenarios or longitudinal testing protocols to account for changes in monitoring behavior and intervention performance over time.

#### Implications for System Design and Policy

Moreover, for dense urban environments, DMS strategies require refinement. The configuration used in our studies did not provide sufficient protection, which argues for stricter urban-specific DMS policies. Warning policies should adapt to gaze history across the drive and, where feasible, to previously experienced events. The proportions of longer single glances capture riskier patterns and enable earlier support. Hence, future DMS could be enhanced by not only tracking cumulative glance durations in specific time windows or extended single glance durations (such as 5 s) but also by monitoring the proportion of single glances exceeding more minor but still critical thresholds (such as 1 s). Adaptive thresholds for such warnings that change with the driver’s evolving behavior and trust level could further improve effectiveness.

Further, the results show that a clear communication of limits improves intervention behavior, timely cues calibrate trust, and reduce delayed takeovers. Thus, announced limits can enhance safety in complex urban settings and do not significantly reduce acceptance (Wiegand et al., 2025). Therefore, we recommend preferring an overcautious warning of potential limits rather than trial-and-error attempts to master situations. Notably, although the absolute collision numbers presented here appear reasonable, the high market penetration rate expected for PAD systems could lead to disproportionate safety consequences if the systems are not designed appropriately for urban environments. These findings have regulatory implications. Current driver monitoring requirements are largely based on general attentional thresholds and may not sufficiently account for the dynamics of repeated PAD exposure in urban environments. Regulatory test protocols may therefore benefit from incorporating repeated-use conditions and event-history effects when evaluating PAD safety. From a system design perspective, these findings suggest that explicit communication of operational limits should be prioritized, as transparent limit communication helps drivers calibrate trust and maintain appropriate monitoring behavior during repeated system use.

#### Future Work

The present work examined driver adaptation during repeated PAD use across urban environments but did not analyze effects at the level of specific urban scenarios. Scenarios such as intersections with crossing traffic or pedestrians or parked vehicle occlusions likely impose different attentional demands. A targeted scenario-specific investigation of driver adaptation would clarify where adaptation harms most and could directly inform interface design and DMS policies for scenario-based strategies. Future work should therefore incorporate and pursue these considerations.

In addition, the present work focused on the influence of system limits during repeated PAD use. Future studies should further examine how the timing and sequence of critical events influence driver adaptation. In particular, the effects of exposure to system failure on trust development, monitoring behavior, and potentially lasting adaptation remain an open question and warrant systematic investigation. Furthermore, to gain a deeper understanding of the drivers’ cognitive processes underlying driver adaptation, future work should investigate how mental models develop across repeated system use.

Finally, individual differences were not analyzed in detail in the present work. Future analyses could investigate whether personal factors, such as driver characteristics or early behavioral indicators, enable the identification of drivers who are more prone to safety-critical outcomes, such as delayed interventions or collisions. Such insights could support adaptive driver monitoring strategies and targeted countermeasures for high-risk users.

## Key Points

• Two multi-session driving simulator studies are presented.
• Repeated use of PAD in urban environments alters drivers’ trust and monitoring behavior.
• Exposure to system limits recalibrates trust and sustains monitoring performance.
• Driver adaptation is highly event-driven as event history shapes attitudes and behavior beyond mere exposure.
• Critical intervention behavior occurs despite the presence of a regulatory-compliant driver monitoring system.

## Supplemental Material

Supplemental Material - Driver Adaptation to Partially Automated Driving in Urban Environments: Effects of Repeated Exposure and System Capabilities on Drivers’ Trust, Monitoring and Response Supplemental Material for Driver Adaptation to Partially Automated Driving in Urban Environments: Effects of Repeated Exposure and System Capabilities on Drivers’ Trust, Monitoring and Response by Elena Malaika Nkusi, Jasmin Verena Schneider, Maximilian Wiegand, and Klaus Bengler in Human Factors.
