# Supplementary material for: Driver Adaptation to Partially Automated Driving in Urban Environments: Effects of Repeated Exposure and System Capabilities on Drivers’ Trust, Monitoring, and Response
Source: Hum Factors. 2026 Jun 18;68(12):1688–711. doi: 10.1177/00187208261460544 (PMC13736686; doi:10.1177/00187208261460544)
Supplement: Supplementary file 1 [file sj-zip-1-hfs-10.1177_00187208261460544.zip › 01_Written System Description.pdf]

WRITTEN SYSTEM DESCRIPTION

"The automated system takes over the longitudinal and lateral control of the vehicle, i.e., you do not have to accelerate, brake, or steer yourself when activated. However, you are responsible for the driving process and must intervene in the event of system failures or if you consider it necessary for other reasons."
