# Supplementary material for: Driver Adaptation to Partially Automated Driving in Urban Environments: Effects of Repeated Exposure and System Capabilities on Drivers’ Trust, Monitoring, and Response
Source: Hum Factors. 2026 Jun 18;68(12):1688–711. doi: 10.1177/00187208261460544 (PMC13736686; doi:10.1177/00187208261460544)
Supplement: Supplementary file 1 [file sj-zip-1-hfs-10.1177_00187208261460544.zip › 02_Exemplary Cover Story.pdf]

EXAMPLE COVER STORY READ OUT LOUD TO THE PARTICIPANTS BEFORE EACH DRIVE

"Please imagine that you have just finished work a bit earlier than usual after a tiring but productive day. It is 2:00 PM, and you are now driving home. The sun is shining, and you are looking forward to a relaxing evening at a restaurant. Your route takes you through the urban environment of your home city, and thanks to the early afternoon timing, you avoid the usual traffic congestion. Make the drive as pleasant as possible for yourself and consider what you would typically do in real life to enjoy the ride. Do you like to listen to the radio, or do you prefer silence and the sounds of traffic?
Please use the partially automated driving system in the vehicle as you would in your own car - there is no right or wrong way to do so.
The automation will follow a predefined route (navigation). Please be aware of your responsibility to monitor both the system and the traffic environment. Additionally, at traffic lights and intersections, there will be blue signs indicating the intended direction if you are driving manually. Please follow these directions and adhere to traffic regulations."
