# Supplementary material for: Driver Adaptation to Partially Automated Driving in Urban Environments: Effects of Repeated Exposure and System Capabilities on Drivers’ Trust, Monitoring, and Response
Source: Hum Factors. 2026 Jun 18;68(12):1688–711. doi: 10.1177/00187208261460544 (PMC13736686; doi:10.1177/00187208261460544)
Supplement: Supplementary file 1 [file sj-zip-1-hfs-10.1177_00187208261460544.zip › 03_ScenarioList.pdf]

1. Signalized intersections incl. lane change to the left lane
2. Signalized intersections incl. decelerating to a slower lead vehicle
3. Signalized intersections incl. turn maneuver
4. Signalized intersection, straight through
5. Give-way (yield) intersection, straight through
6. Priority-for-ego intersections, straight through
7. Y-fork junction with left turn
8. Y-fork junction with right turn and crossing cyclist
9. Pedestrian at a zebra crossing
10. Passing emergency vehicles
11. Cyclists on bicycle advisory lanes at intersections, crossing interaction
12. Obstacle in ego lane (stopped postal van)
13. Work zone, straight through
14. Construction site in ego lane
15. City expressway
16. Residential area with vehicles parked on both sides of the street
17. Transitions between road types
18. Driving under a pedestrian bridge
19. Driving on one-way streets
20. Driving on two-lane roads
21. Driving on four-lane roads

*Some of the scenarios were experienced multiple times and in different variations per drive repetition.
