# Supplementary material for: Driver Adaptation to Partially Automated Driving in Urban Environments: Effects of Repeated Exposure and System Capabilities on Drivers’ Trust, Monitoring, and Response
Source: Hum Factors. 2026 Jun 18;68(12):1688–711. doi: 10.1177/00187208261460544 (PMC13736686; doi:10.1177/00187208261460544)
Supplement: Supplementary file 1 [file sj-zip-1-hfs-10.1177_00187208261460544.zip › 04_Questionnaire_Items.pdf]

QUESTIONNAIRES

Code	Question	Response Scale
Demo_Q1	Participant Code	Free text/code
Demo_Q2	What is your gender?	Single choice (female/male/diverse)
Demo_Q3	How old are you?	Numeric (age)
Demo_Q4	How long have you held a valid driver's license?	Single choice (<2 years / >2 years)
Demo_Q5	How often do you drive a car?	Single choice (4 levels of frequency)
PreExp_Q0	Experience with assistance systems	Categorical (5 levels of experience)
PreExp_Q1	Cruise Control	Categorical (5 levels of experience)
PreExp_Q2	Adaptive Cruise Control (ACC)	Categorical (5 levels of experience)
PreExp_Q3	Traffic Jam Assist	Categorical (5 levels of experience)
PreExp_Q4	Lane Departure Warning	Categorical (5 levels of experience)
PreExp_Q5	Lane Keeping Assist (LKA)	Categorical (5 levels of experience)
PreExp_Q6	ACC + LKA	Categorical (5 levels of experience)
PreExp_Q7	Have you previously used a driving simulator?	Yes/No
PreExp_Q10	Did you experience discomfort (e.g., headache) while using the simulator?	Yes/No
TiA_Q1	The system is capable of assessing situations correctly.	Likert 5-point (Strongly agree – Strongly disagree)
TiA_Q2	I will always be aware of the system's state.	Likert 5-point (Strongly agree – Strongly disagree)
TiA_Q3	One should be cautious with unfamiliar automated systems.	Likert 5-point (Strongly agree – Strongly disagree)
TiA_Q4	The system works reliably.	Likert 5-point (Strongly agree – Strongly disagree)
TiA_Q5	The system reacts unpredictably.	Likert 5-point (Strongly agree – Strongly disagree)
TiA_Q6	I trust the system.	Likert 5-point (Strongly agree – Strongly disagree)
TiA_Q7	A system failure is likely.	Likert 5-point (Strongly agree – Strongly disagree)
TiA_Q8	I will be able to understand what is happening.	Likert 5-point (Strongly agree – Strongly disagree)
TiA_Q9	I tend to trust rather than mistrust such systems.	Likert 5-point (Strongly agree – Strongly disagree)
TiA_Q10	The system can handle complex tasks.	Likert 5-point (Strongly agree – Strongly disagree)
TiA_Q11	I can rely on the system.	Likert 5-point (Strongly agree – Strongly disagree)
TiA_Q12	The system might make occasional mistakes.	Likert 5-point (Strongly agree – Strongly disagree)
TiA_Q13	It is difficult to predict the system's next move.	Likert 5-point (Strongly agree – Strongly disagree)
TiA_Q14	Automated systems generally work well.	Likert 5-point (Strongly agree – Strongly disagree)
TiA_Q15	I am confident in the system's capabilities.	Likert 5-point (Strongly agree – Strongly disagree)
ATI_Q1	I like to explore new technologies.	Likert 5-point (Strongly agree – Strongly disagree)
ATI_Q2	When I have a problem with a technical device, I try to solve it on my own.	Likert 5-point (Strongly agree – Strongly disagree)
ATI_Q3	I enjoy spending time becoming acquainted with a new technical system.	Likert 5-point (Strongly agree – Strongly disagree)
ATI_Q4	I find it difficult to deal with technical systems.	Likert 5-point (Strongly agree – Strongly disagree)
ATI_Q5	I enjoy testing the functions of new technical systems.	Likert 5-point (Strongly agree – Strongly disagree)
ATI_Q6	I try to understand how a technical system exactly works.	Likert 5-point (Strongly agree – Strongly disagree)
ATI_Q7	It is enough for me that a technical device works; I do not care how or why.	Likert 5-point (Strongly agree – Strongly disagree)
ATI_Q8	I am very curious about new technologies.	Likert 5-point (Strongly agree – Strongly disagree)
ACPT_1	Useful – Useless	5-point bipolar scale
ACPT_2	Pleasant – Unpleasant	5-point bipolar scale
ACPT_3	Good – Bad	5-point bipolar scale
ACPT_4	Nice – Annoying	5-point bipolar scale
ACPT_5	Efficient – Inefficient	5-point bipolar scale
ACPT_6	Helpful – Irritating	5-point bipolar scale
ACPT_7	Desirable – Undesirable	5-point bipolar scale
ACPT_8	Stimulating – Boring	5-point bipolar scale
DMS_Q1	How appropriate did you find the warnings from the DMS?	Likert 5-point (1 = very inappropriate; 5 = very appropriate)
DMS_Q2	Optional: Please briefly justify your assessment.	Free text
