# Supplementary material for: Driver Adaptation to Partially Automated Driving in Urban Environments: Effects of Repeated Exposure and System Capabilities on Drivers’ Trust, Monitoring, and Response
Source: Hum Factors. 2026 Jun 18;68(12):1688–711. doi: 10.1177/00187208261460544 (PMC13736686; doi:10.1177/00187208261460544)
Supplement: Supplementary file 1 [file sj-zip-1-hfs-10.1177_00187208261460544.zip › 06_Statistical_Reports_Study1.pdf]

<u>Trust</u>

rmANOVA

	Effect	df	MSE	F	ges	pes	p.value
1	Drive_rep	2.52, 52.86	0.62	1.59	.048	.070	.208
2	PrePost	1, 21	0.12	2.49	.006	.106	.130
3	Drive_rep:PrePost	3.07, 64.46	0.13	9.05 ***	.071	.301	<.001

Post-hoc tests (holm corrected)

PrePost = before:

contrast	estimate	SE	df	t.ratio	p.value	d
X1 - X2	-0.30556	0.1920	21	-1.589	0.7619	-0.3388
X1 - X3	-0.29798	0.1570	21	-1.895	0.5758	-0.4040
X1 - X4	-0.41919	0.0958	21	-4.375	0.0024	-0.9328
X1 - X5	-0.54167	0.1110	21	-4.863	0.0008	-1.0367
X2 - X3	0.00758	0.1410	21	0.054	1.0000	0.0114
X2 - X4	-0.11364	0.1550	21	-0.734	1.0000	-0.1565
X2 - X5	-0.23611	0.1520	21	-1.556	0.7619	-0.3318
X3 - X4	-0.12121	0.1430	21	-0.848	1.0000	-0.1808
X3 - X5	-0.24369	0.1350	21	-1.810	0.5927	-0.3858
X4 - X5	-0.12247	0.0856	21	-1.431	0.7619	-0.3052

PrePost = after:

contrast	estimate	SE	df	t.ratio	p.value	d
X1 - X2	-0.04545	0.2130	21	-0.214	1.0000	-0.0455
X1 - X3	-0.08207	0.1820	21	-0.452	1.0000	-0.0963
X1 - X4	-0.25000	0.1500	21	-1.671	0.8761	-0.3563
X1 - X5	0.23359	0.1600	21	1.457	0.8761	0.3107
X2 - X3	-0.03662	0.1550	21	-0.236	1.0000	-0.0503
X2 - X4	-0.20455	0.1240	21	-1.644	0.8761	-0.3505
X2 - X5	0.27904	0.1780	21	1.568	0.8761	0.3343
X3 - X4	-0.16793	0.1370	21	-1.226	0.9353	-0.2613
X3 - X5	0.31566	0.1480	21	2.130	0.4063	0.4542
X4 - X5	0.48359	0.1240	21	3.907	0.0081	0.8330

PEORT

rmANOVA

	Effect	df	MSE	F	ges	pes	p.value
1	Drive_rep	2.32, 48.68	103.39	1.35	.025	.060	.270

PEC

rmANOVA

	Effect	df	MSE	F	ges	pes	p.value
1	Drive_rep	2.39, 50.19	7.49	3.21 *	.062	.133	.040

Post-hoc tests (holm corrected)

contrast	estimate	SE	df	t.ratio	p.value	d
X1 - X2	0.1917	0.535	21	0.358	1.0000	0.0763
X1 - X3	0.1188	0.917	21	0.130	1.0000	0.0276
X1 - X4	1.6157	0.480	21	3.365	0.0293	0.7174
X1 - X5	1.5302	0.570	21	2.686	0.1006	0.5726
X2 - X3	-0.0729	0.759	21	-0.096	1.0000	-0.0205
X2 - X4	1.4240	0.522	21	2.729	0.1006	0.5819
X2 - X5	1.3385	0.454	21	2.950	0.0688	0.6290
X3 - X4	1.4969	0.781	21	1.918	0.4131	0.4089
X3 - X5	1.4114	0.790	21	1.786	0.4430	0.3807
X4 - X5	-0.0855	0.310	21	-0.276	1.0000	-0.0588
